# Late chronic local inflammation, synaptic alterations, vascular remodeling and arteriovenous malformations in the brains of male rats exposed to repetitive low-level blast overpressures

**DOI:** 10.1186/s40478-023-01553-6

**Published:** 2023-05-12

**Authors:** Miguel A. Gama Sosa, Rita De Gasperi, Dylan Pryor, Georgina S. Perez Garcia, Gissel M. Perez, Rania Abutarboush, Usmah Kawoos, Seth Hogg, Benjamin Ache, Allison Sowa, Timothy Tetreault, Merina Varghese, David G. Cook, Carolyn W. Zhu, Susan J. Tappan, William G. M. Janssen, Patrick R. Hof, Stephen T. Ahlers, Gregory A. Elder

**Affiliations:** 1 General Medical Research Service, James J. Peters Department of Veterans Affairs Medical Center, 130 West Kingsbridge Road, Bronx, NY 10468 USA; 2 Department of Psychiatry, Icahn School of Medicine at Mount Sinai, One Gustave Levy Place, New York, NY 10029 USA; 3 Friedman Brain Institute, Icahn School of Medicine at Mount Sinai, New York, NY 10029 USA; 4 Research and Development Service, James J. Peters Department of Veterans Affairs Medical Center, 130 West Kingsbridge Road, Bronx, NY 10468 USA; 5 Department of Neurology, Icahn School of Medicine at Mount Sinai, One Gustave Levy Place, New York, NY 10029 USA; 6 Nash Family Department of Neuroscience, Icahn School of Medicine at Mount Sinai, New York, NY 10029 USA; 7 Department of Neurotrauma, Operational and Undersea Medicine Directorate, Naval Medical Research Center, 503 Robert Grant Avenue, Silver Spring, MD 20910 USA; 8 The Henry M. Jackson Foundation for the Advancement of Military Medicine, Inc., Bethesda, MD USA; 9 Micro Photonics, Inc, 1550 Pond Road, Suite 110, Allentown, PA 18104 USA; 10 MBF Bioscience LLC, 185 Allen Brook Lane, Williston, VT 05495 USA; 11 Geriatric Research Education and Clinical Center, VA Puget Sound Health Care System, 1660 S Columbian Way, Seattle, WA 98108 USA; 12 Department of Medicine, University of Washington, 1959 NE Pacific St, Seattle, WA 98195 USA; 13 Mount Sinai Alzheimer’s Disease Research Center and the Ronald M. Loeb Center for Alzheimer’s Disease, Icahn School of Medicine at Mount Sinai, New York, NY 10029 USA; 14 Department of Geriatrics and Palliative Care, Icahn School of Medicine at Mount Sinai, New York, NY 10029 USA; 15 Neurology Service, James J. Peters Department of Veterans Affairs Medical Center, 130 West Kingsbridge Road, Bronx, NY 10468 USA

**Keywords:** Animal model, Blast, Brain, Chronic, Vascular, Neurovascular unit, Rat, Vascular pathology, Microglia, Synapse, Neuroinflammation

## Abstract

In the course of military operations in modern war theaters, blast exposures are associated with the development of a variety of mental health disorders associated with a post-traumatic stress disorder-related features, including anxiety, impulsivity, insomnia, suicidality, depression, and cognitive decline. Several lines of evidence indicate that acute and chronic cerebral vascular alterations are involved in the development of these blast-induced neuropsychiatric changes. In the present study, we investigated late occurring neuropathological events associated with cerebrovascular alterations in a rat model of repetitive low-level blast-exposures (3 × 74.5 kPa). The observed events included hippocampal hypoperfusion associated with late-onset inflammation, vascular extracellular matrix degeneration, synaptic structural changes and neuronal loss. We also demonstrate that arteriovenous malformations in exposed animals are a direct consequence of blast-induced tissue tears. Overall, our results further identify the cerebral vasculature as a main target for blast-induced damage and support the urgent need to develop early therapeutic approaches for the prevention of blast-induced late-onset neurovascular degenerative processes.

## Introduction

Veterans of modern day wars often have an overlapping history of blast-induced traumatic brain injury (TBI) and post-traumatic stress disorder (PTSD) [23]. Although many veterans who suffer blast-related TBIs experience improvement of symptoms, others exhibit chronic post-concussive and mental health-related symptoms, which often worsen over time. This decline is driven mainly by worsening PTSD and depression that are largely refractory to therapy [54]. Several aspects of blast-induced TBI have been modeled in animal systems, which exhibit PTSD-related traits including anxiety, enhanced acoustic startle, altered fear learning and impaired cognition that develop over time [66, 76]. The mechanisms by which blast exposure predisposes the individual to PTSD are expected to affect the integrity and function of brain structures involved in the neurobiology of the disorder [55].

Acute and chronic cerebral vascular degeneration is a well-established component of blast injury in humans and animal models [1, 24, 27, 28, 31, 40, 41, 52, 59, 69, 70, 72, 75, 79]. Observations from the recent conflicts in Iraq, Afghanistan and Ukraine, have shown that high-pressure blast waves from explosive devices including improvised explosive devices (IEDs), artillery ammunition, and mortar shells cause extensive multi-organ trauma, including in the central nervous system (CNS), and severe systemic vascular injury including edema, intracranial hemorrhage, and vasospasm along with reduced cerebral perfusion and altered contractile properties of large arteries [70, 94].

As in humans, acute high-level blast exposure in rodent models has a prominent hemorrhagic component that includes venous hemorrhages [24]. Acute blast exposure has been associated with reduced cerebral blood flow, increased vascular permeability, blood–brain barrier (BBB) breakdown and other vascular injuries including apoptosis of vascular structural elements, capillary strictures, vascular occlusion, BBB disruption, vascular smooth muscle phenotypic change, vasospasms, vascular rupture, breakdown of the choroid plexus, reduced dilator responses to decreased intravascular pressure, reduced cerebral perfusion, increased cerebral vascular resistance, perivascular astrocytic alterations and decreased perivascular astrocytic coverage [1, 2, 27–31, 40, 43, 45, 47, 51, 58, 59, 71, 93]. Blast exposure in mice produces acute microlesions in the BBB that are associated with aberrant expression of phosphorylated tau protein [41, 58].

The acute vascular injuries described above are followed by development of a secondary pathology characterized by perivascular astrocytic degeneration, luminal collapse, altered neurovascular interactions, vascular structural disruptions, extracellular matrix (ECM) remodeling, intraluminal astrocytic processes, vascular smooth muscle degeneration, vascular occlusion by CD34^+^ progenitor cells, vasoconstriction, generalized vascular attenuation, enlarged paravascular spaces, perivascular inflammation, aneurysm formation and vascular leakage [29–31]. Blast-induced vasospasm was suggested to initiate a phenotypic switch in vascular smooth muscle cells that causes long-term vascular remodeling [39]. In rats, evolution of blast-induced cognitive and behavioral phenotypes seem to overlap with the development of chronic cerebral vascular degenerative processes. [27–29] and recent transcriptomic studies have suggested that an inflammatory signature develops over the same time frame [33].

The present research is an extension of a previous report on the late effects of repetitive blast exposure in a male rat model of blast-induced mild TBI (mTBI; 13 months post exposure) [31]. Here we present evidence linking vascular degeneration with dendritic degeneration, synapse alterations, and inflammation. We also present evidence that blast-induced tissue shearing induces the formation of vascular malformations. All together blast-induced vascular alterations are at the core of the neuropathological events that result in blast-related symptomatology.

## Materials and methods

### Animals

All studies were reviewed and approved by the Institutional Animal Care and Use Committees of the Walter Reed Army Institute of Research/Naval Medical Research Center and the James J. Peters VA Medical Center. Studies were conducted in compliance with the Public Health Service policy on the humane care and use of laboratory animals, the NIH Guide for the Care and Use of Laboratory Animals, and all applicable Federal regulations governing the protection of animals in research. Young adult male Long-Evans hooded rats (250–350 g) of 8 weeks of age were obtained from Charles River Laboratories International (Wilmington, MA, USA) and blast exposed at 10 weeks of age. Animals were housed at a constant 70–72 °F temperature on a 12:12 h light cycle. All rats were kept individually in standard clear plastic cages with bedding and nesting paper. Access to food and water was ad libitum.

### Blast overpressure exposure

Blast exposures were performed with the Walter Reed Army Institute of Research shock tube located at the Naval Medical Research Center (Silver Springs, MD, USA). This instrument has been used in our prior studies to deliver blast overpressure injuries to rats [3, 8–10, 18, 22, 28, 29, 31]. Rats were anesthetized with isoflurane and randomly assigned to sham or blast-exposed groups. Animals were placed in a plastic cone (Decapicone, Braintree Scientific, MA, USA) and secured in a basket inside the shock tube in the facing orientation. For blast exposure, the head was facing the source of compressed air without any body shielding, resulting in a full body exposure to the blast wave. The physical characteristics of the blast wave and further details of the blast exposure have been described elsewhere [3]. Blast-exposed animals received a total of three 74.5-kPa (10.8 psi) exposures, with one exposure administered daily for 3 consecutive days. Control animals were anesthetized and placed in the blast tube but not subjected to a blast exposure. Within 10 days after the last blast exposure, the animals were transferred to the James J. Peters VA Medical Center (Bronx, NY, USA) where all other procedures were performed. Control and experimental animals were euthanized at 13 months post-exposure to analyze the chronic neuropathological effects of blast exposure in the brain.

The animals analyzed in this study (4 blast-exposed and 5 sham-exposed animals) were part of a larger cohort that was previously subjected to behavioral analysis [64]. Additional animals used for western blot (n = 5/group) analysis were from the same cohort.

### Visual reconstruction of the cerebral angioarchitecture

Rats were anesthetized with 150 mg/kg ketamine and 30 mg/kg xylazine and transcardially perfused first with 60 ml of 10 µg/ml heparin in phosphate-buffered saline (PBS), pH 7.2, followed by 250 ml of a 30% solution of the Brite Vu Special Projects contrast agent supplemented with its enhancer (Scarlet Imaging, Murray UT, USA) maintained at 65 °C. The perfused animals were chilled by total immersion in an ice-water bath for 2 h to gel the intravascular contrasting agent. Brains were post-fixed overnight in 4% paraformaldehyde in PBS and maintained in sterile PBS at 4 °C. Brains were scanned at a 7.5-µm voxel size using 60 kV, 166 µA X-ray settings and a 0.25-mm aluminum filter with exposure time of 508 ms per frame, with 3 frames averaged at each projection angle with a Bruker SkyScan 1272 micro-CT (Micro Photonics, Allentown, PA, USA). Three-dimensional reconstruction and morphological profiling of the cerebral vasculature were performed with the Bruker’s NRecon software or the Vesselucida 360 software (v2018.1.1, MBF Bioscience LLC, Williston, VT, USA) using data obtained from the micro-CT scans and reconstructing the respective three-dimensional vascular networks. For hippocampal regional quantitative analyses, automatic reconstruction of the vasculature in a 2.25-mm optical section was performed with the Vesselucida 360 software (approximate coordinates interaural 7.2–4.0 mm). Tracing of the hippocampal region was based on corresponding images in a rat brain atlas [62]. A voxel scooping algorithm was applied with trace and seed sensitivity set to 90, high seed density with refine filter set to 1, and maximum gap tolerance. No manual editing was performed. The parameters determined were total vascular length, diameter and volume.

### General histology and immunohistochemistry

Coronal sections (50 µm thickness) of the micro-CT scanned brains were prepared with a VT1000S Vibratome (Leica Biosystems, Buffalo Grove, IL, USA). General histological evaluation was performed on hematoxylin & eosin (H&E)-stained sections. For immunohistochemistry, floating sections were blocked with 10% normal goat serum in 50 mM Tris HCl, pH 7.6, 0.15 M NaCl, 0.3% Triton-X-100 and incubated overnight with the primary antibodies diluted in blocking solution at room temperature. After washing with PBS (6 times for 10 min each), sections were incubated with the appropriate Alexa Fluor (488, 568 and 647)-conjugated secondary antibodies (1:300, ThermoFisher, Waltham MA, USA) in blocking solution for 2 h. After washing with PBS (6 times for 10 min each), the sections were mounted with Fluorogel mounting medium (Electron Microscopy Sciences, Hatfield, PA, USA). To visualize nuclei, sections were incubated in 0.1 µg/ml DAPI (4',6-diamidine-2’-phenylindole dihydrochloride) in PBS in the next to last PBS wash. The primary antibodies included a rat monoclonal anti-glial fibrillary acidic protein (GFAP; 1:500, clone 2.2B10, gift of Dr. Virginia Lee, University of Pennsylvania, Philadelphia PA, USA), mouse monoclonal anti-neuronal class III β-Tubulin (TUJ1; 1:300, MMS 435P, RRID: AB_2313773, Covance, Emeryville, CA, USA) and rabbit anti‐ionized calcium‐binding adaptor molecule 1 (Iba1; 1:300, 019–19,741, RRID: AB_839504, Fujifilm Wako Pure Chemical, Osaka, Japan). Vascular staining was performed with anti-collagen type IV antibodies (rabbit anti-rat collagen type IV, 1:300, ab6586, RRID: AB_305584, Abcam, Cambridge, MA, USA) without pepsin pre-treatment to visualize ECM remodeling [26, 32]. For TUNEL (terminal deoxynucleotidyltransferase-mediated dUTP nick-end labeling) staining, sections were washed in Tris-buffered saline (TBS), permeabilized with 0.1% Triton X-100 in TBS for 1 h and washed extensively with TBS. End labeling of DNA with fluorescein-dUTP was performed using a commercial kit (Roche, Indianapolis, IN, USA). After several washes with PBS, the sections were blocked and stained with antibodies against GFAP and ionized calcium-binding adaptor molecule (Iba1) as described above.

### Electron microscopy

Electron microscopy was performed using our protocols optimized to study the ultrastructure of the vasculature [31]. Anesthetized rats were transcardially perfused with ice-cold 2.0% glutaraldehyde, 2% paraformaldenyde, 0.1 M sodium phosphate (pH 7.2). Brains were removed, postfixed in the same fixative as above and stored at 4 °C until further processing. Fixed brains were placed on a rat brain slicer matrix, and coronal slices containing the frontal cortex were excised. Sections were washed in 0.1 M sodium cacodylate buffer, pH 7.2 and postfixed with 1% osmium tetroxide in 0.1 M sodium cacodylate buffer, pH 7.2. Sections were washed again in cacodylate buffer, dehydrated through graded ethanol (70–100%) and propylene oxide series, and resin-infiltrated with Epon (Electron Microscopy Sciences). Material was polymerized in a vacuum oven at 60 °C for 48 h. Semi-thin (1 µm) toluidine blue-stained sections were used to identify regions of interest. Ultrathin sections (80 nm) were cut with a diamond knife on a Leica UCT ultramicrotome and mounted on copper grids using a Coat-Quick adhesive pen (Electron Microscopy Sciences). Sections were counterstained with uranyl acetate and lead citrate. Frontal cortical sections were imaged on a Hitachi 7700 electron microscope (Hitachi, Ltd., Tokyo, Japan) and photographed with an Advantage CCD camera (Advanced Microscopy Techniques, Danvers, MA, USA). Image brightness and contrast were adjusted using Adobe Photoshop 2022 software (version 23.4.1; Adobe, San Jose, CA, USA).

### Western blotting of synaptic proteins

Dissected hippocampi from control and blast-exposed rats (n = 5, per group) were lysed in 10 mM NaPO_4_, pH 7.4, 150 mM NaCl, 2 mM EDTA, 1% Triton X-100, 0.5% sodium deoxycholate, and 1% sodium dodecyl sulfate (SDS) supplemented with protease and phosphatase inhibitor cocktails 2 and 3 (Sigma-Aldrich, St Louis, MO, USA). The lysates were centrifuged at 15,000 × *g* for 15 min, and the protein concentration in the supernatants was determined with the BCA reagent (ThermoFisher) according to the manufacturer’s protocol. Proteins (50 μg) were separated by SDS–polyacrylamide gel electrophoresis (PAGE), and the gels blotted onto polyvinylidene difluoride (PVDF) membranes. The membranes were blocked in a solution containing 50 mM Tris–HCl, pH 7.6, 0.15 M NaCl (TBS) and 0.5% non-fat dry milk and incubated overnight at 4 °C with primary antibody diluted in blocking solution. Membranes were incubated with the appropriate horseradish peroxidase-conjugated secondary antibody (1:5000–1:10,000, Cytiva, Marlborough, MA, USA) in blocking solution, and the bands visualized with the ECL Prime Western Blot detection reagent (Cytiva). The blots were imaged with the Amersham Imager 800 (Cytiva), and bands quantitated with Image QuantTL software (Cytiva). The following primary antibodies and dilutions were used: rabbit monoclonal antibodies against postsynaptic density protein 95 (PSD95, 1:1000, #3450, RRID: AB_2292883, clone D27E11), and spinophilin (1:1000, #14,136, RRID: AB_2572261, clone E1E7R) from Cell Signaling Technology (Danvers, MA, USA); and synaptophysin (1:1400, #04–1019, RRID: AB_2286949, clone YE269, Millipore, Temecula, CA, USA).

### Statistical analyses

Statistical differences were assessed with unpaired *t*-tests using Prism 9.0 software (GraphPad, La Jolla, CA, USA). Wilcoxon Rank Sum Test was used to examine differences in vascular length, diameter and volume between non-affected and affected sides (SAS, Cary, NC,USA). Fisher's exact test was used to analyze the distribution of vessel diameters. Statistical significance was set at an α level of 0.05.

## Results

### Novel object recognition testing of animals analyzed in this study

**Fig. 1:**
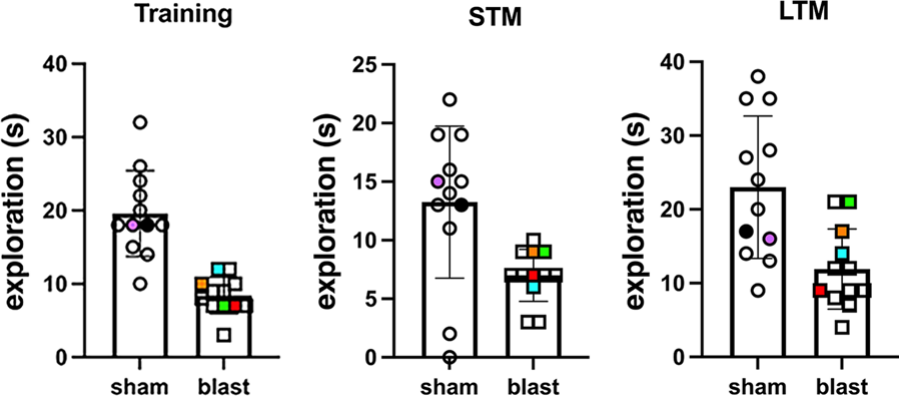
Novel Object Recognition (NOR) testing of animals used in this study. Blast-exposed (n = 12) and control (n = 12) rats from cohort 2 in [64] were tested in a NOR task 41 weeks post-blast exposure. Shown is total exploration time (s) during the training session and time spent exploring the novel object during short-term memory (STM, 1 h after training) and long-term memory (LTM, 24 h after training) testing. 5 control and 4 blast-exposed rats from the above cohort 2 were perfused at 13 months after blast exposure with the Brite Vu Special Projects contrast agent. Quantitative analysis of 9 vascular parameters derived from the micro CT scans of these rats were previously presented [31]. Animals indicated in color were used to illustrate additional aspects of the vascular pathology presented in this study. Blast-exposed animals: blue square, Figs. 2 and 19; orange square, Figs. 3–4; red square, Figs. 5–13 and 14c; green square, Figs. 16–18. Controls: black circle, Figs. 9e and 14c (synaptophysin); purple circle, Fig. 14c (PSD95 and spinophilin)

Blast-exposed and control rats used in this study were part of a cohort that was subjected to behavioral testing (cohort 2 in [64]). Rats at 41 weeks post-exposure showed deficits in the Novel Object Recognition (NOR) task [64]. Figure 1 shows the NOR results for the entire cohort. Specific blast-exposed and sham control animals illustrated in this study are identified by colored squares and colored dots, respectively.

### Late-onset perivascular inflammation is present 13 months post blast exposure

**Fig. 2:**
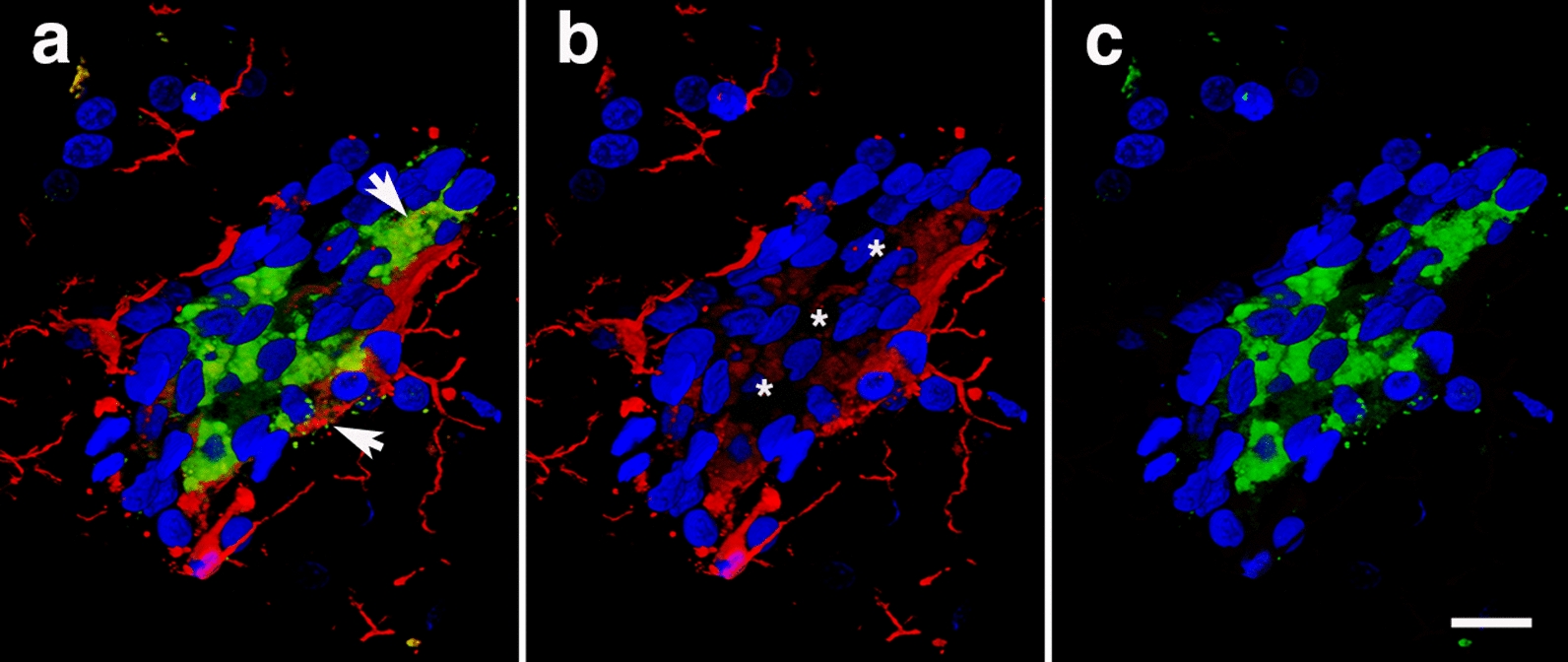
Activated perivascular microglia. Luminal view of a patch of perivascular M1 activated microglia (expressing MHCII) [31]. **a**, Merged images; **b**, Iba1 immunostaining (microglia, red); **c**, MHCII immunostaining (activated M1 microglia, green). Arrows in **a** show an apoptotic activated microglial cell (green) and a degenerating perivascular microglial cell (red). Asterisks in **b** indicate the lumen. Scale, 20 µm

**Fig. 3:**
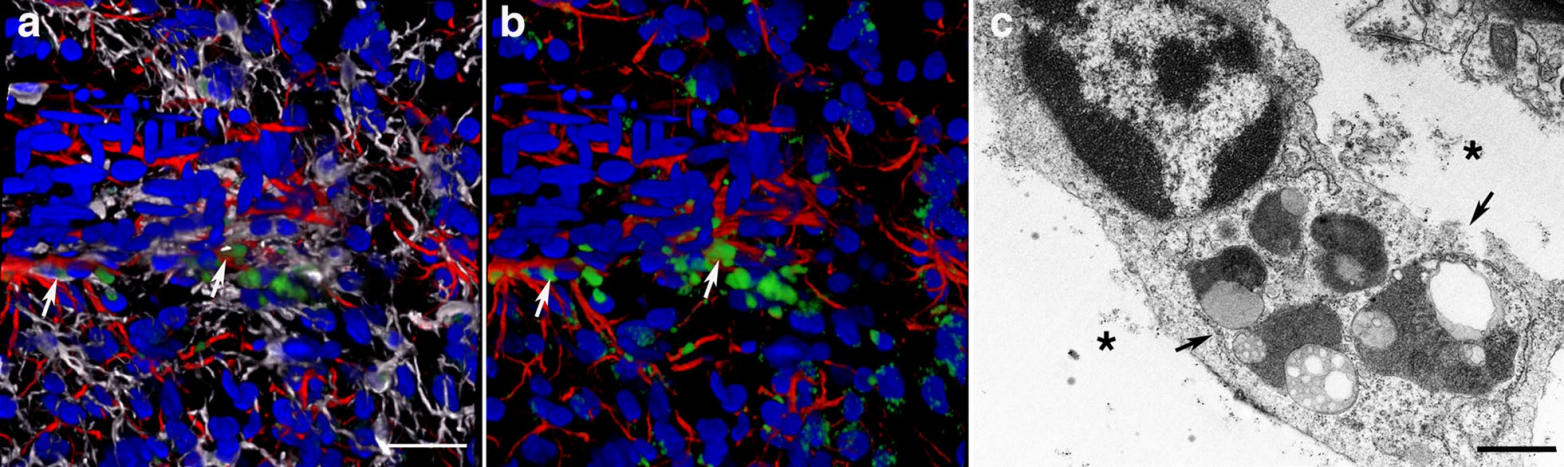
Periarterial apoptotic microglia and astrocytes. **a** and **b** show a cerebral artery (identified by its longitudinal and transverse smooth muscle nuclei) surrounded by a patch of activated microglia and stained with TUNEL. Microglia (Iba1, white), astrocytes (GFAP, red), and nuclei (DAPI, blue). Note the presence of apoptotic bodies (green, arrows) in activated unramified microglia and perivascular astrocytes. **c**, Electron microscopy of an activated microglial cell within an enlarged paravascular space (asterisks) and harboring secondary lysosomes and residual bodies (arrows). Scale, 30 µm in **a** and **b**, and 1 µm in **c**

**Fig. 4:**
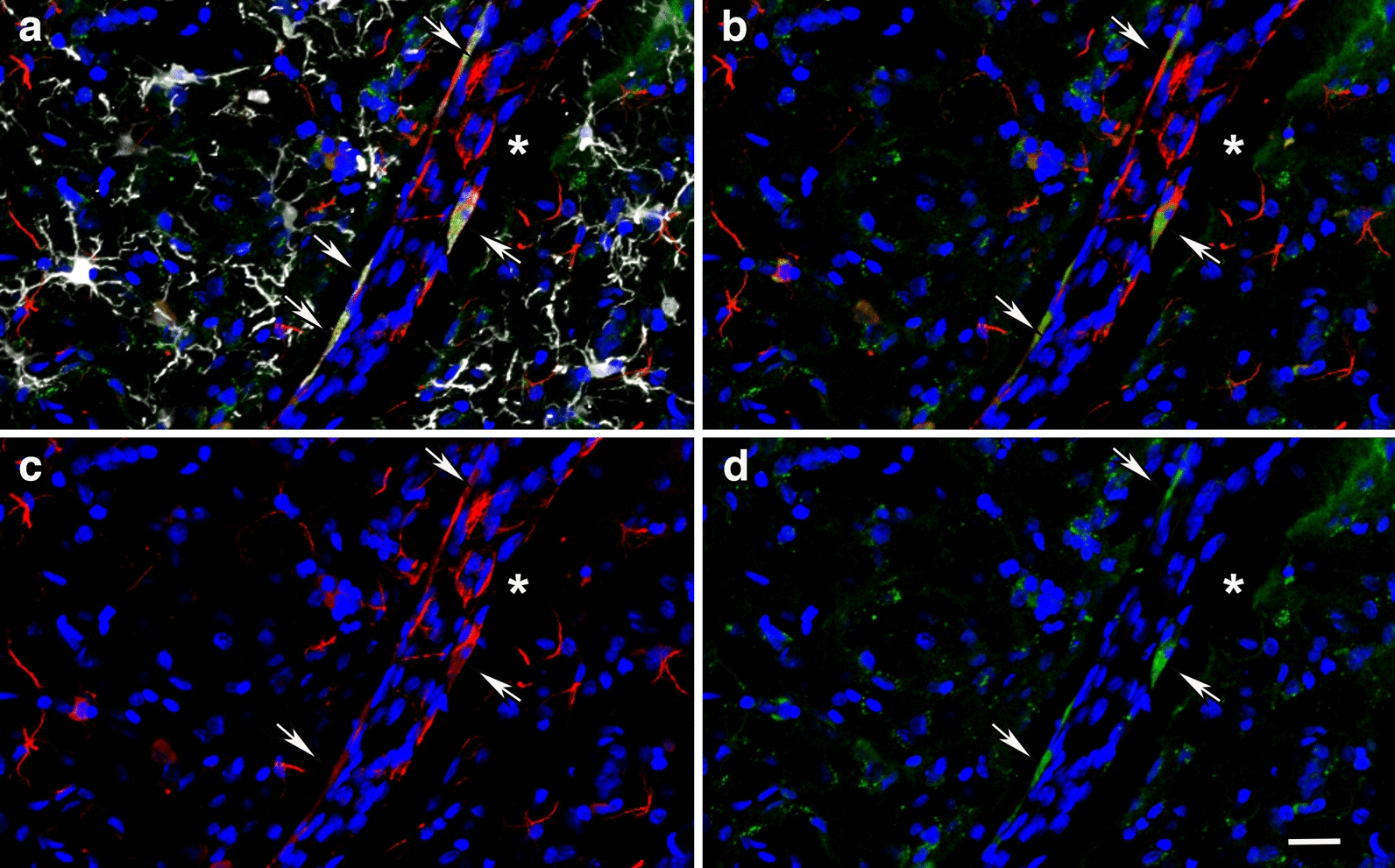
Apoptotic microglia intimately associated with perivascular astrocytes. Arrows show unramified TUNEL-positive microglia with ameboid morphology in close contact with perivascular astrocytes (**a-d**). Iba1 (white), TUNEL (green), GFAP (red), and DAPI (blue) staining. Asterisks (*) denote enlarged paravascular space. Scale, 40 µm

We previously reported that brains of blast-exposed animals (13 months post-exposure) present attenuated vasculature with enlarged paravascular spaces and loss or degeneration of perivascular astrocytes [31]. We also reported the presence of perivascular inflammation in the brains of blast-exposed animals (13 months post-exposure) as we observed patches of M1 activated microglia associated with medium and large vessels [31]. Figure 2 shows examples of perivascular Iba1/MHCII-immunoreactive cells (type 1 inflammation) with membrane blebs characteristic of cells undergoing apoptosis (arrows in panel a). TUNEL staining was used to further investigate apoptotic DNA breakage. TUNEL-positive staining was mostly found in perivascular microglia and astrocytes. For example, Fig. 3 shows the presence of TUNEL-positive periarterial astrocytes and activated microglia that were frequently found in contact with neighboring perivascular astrocytes in vessels with enlarged paravascular spaces (Fig. 4). Electron microscopy of microglia within enlarged paravascular spaces also identified cells undergoing active phagocytosis based on the presence of secondary lysosomes and residual bodies (Fig. 3c, arrows).

### Vascular degeneration is associated with local gliosis and loss of neuronal cells

**Fig. 5:**
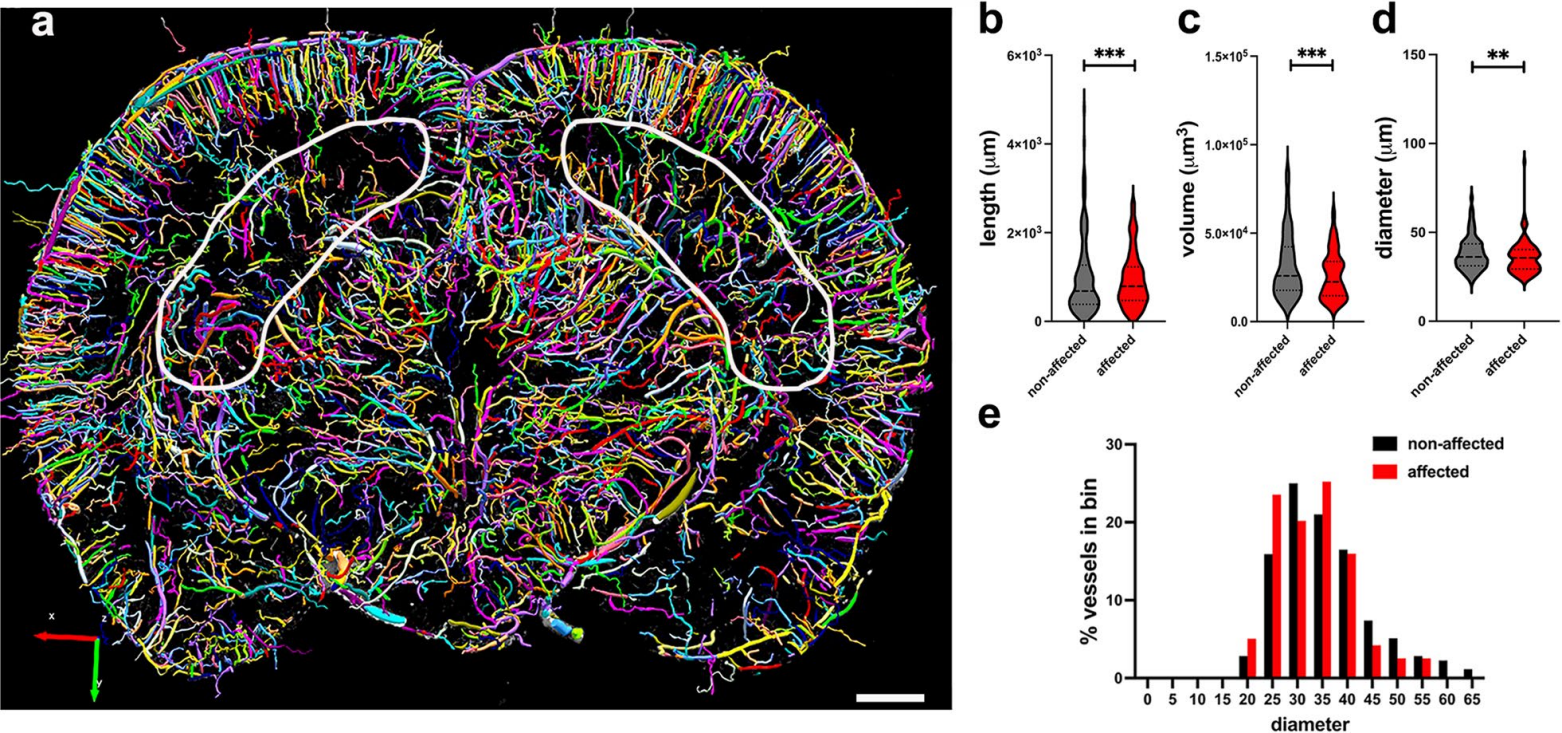
Hippocampal vascular alterations associated with local inflammation. Stereological comparison of the ipsilateral (inflammation present, left side in panel **a**) and contralateral hippocampal (no inflammation present, right side in panel **a**) vasculatures in the brain of a blast-exposed rat. **a**, Optical coronal  sections (2.25 mm-thick) showing color-coded reconstructed cerebral vasculature from a micro-CT scan (interaural 7.20–4.95 mm). Hippocampal vasculature is indicated within the contours. Vasculature analysis was performed with the Vesselucida software (179 vessels analyzed in the non-affected side and 119 in the affected side). **b**-**d**, vessels length (**b**), volume (**c**) and diameter (**d**). **e**, percent vessels distribution relative to diameter. **b**-**d**, data were analyzed using the Wilcoxon Rank Sum test; **e**, Fisher's exact test. Asterisks indicate statistically significant differences between groups (**, *p* < 0.01; ***, p < 0.001). Scale, 1 mm

The hippocampal vasculature was quantitatively analyzed in a 2.25-mm micro-CT optical section (interaural 7.20–4.95 mm) from a blast-exposed animal showing inflammation (Fig. 5). Data were analyzed with the Wilcoxon Rank Sum test and showed a significant decrease in vascular length, diameter and volume on the hippocampal side showing inflammation (*p* < 0.01, Fig. 5b–d). Analysis of vessel distribution according to diameter (Fig. 5e) suggested that the affected vasculature associated with inflammation was more likely to be in bins of smaller diameter (Fisher's exact test, *p* < 0.0001).

**Fig. 6:**
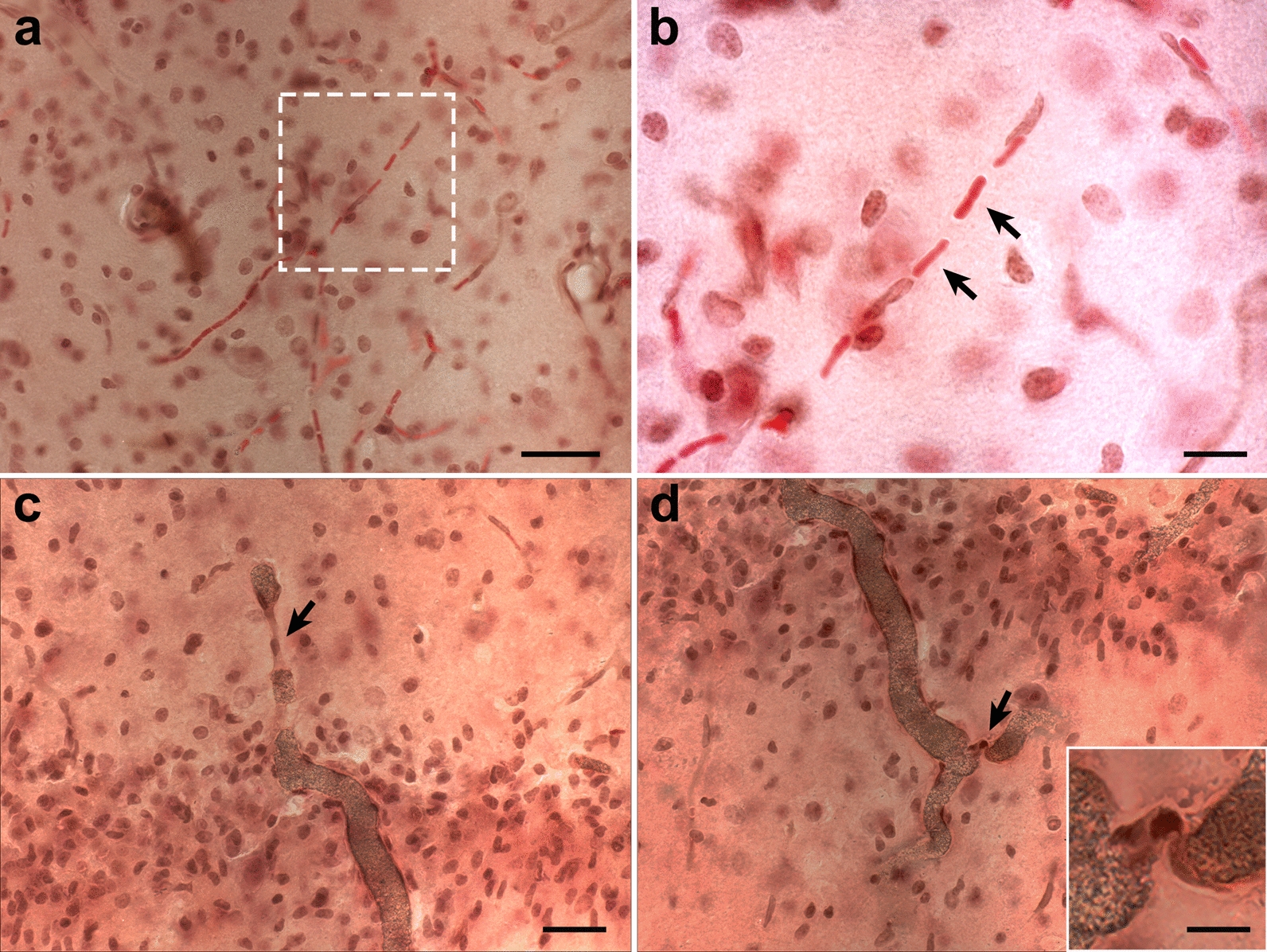
Vascular constrictions in the hippocampal region affected by local inflammation. **a**, Constricted microvasculature with elongated red blood cells in an H&E-stained section. Note the lack of the dark Brite Vu contrast agent used to perfuse this animal within these vessels. **b**, Higher magnification of microvessel indicated in the box from **a**. Arrows indicate the highly elongated boxcar-like appearance of red blood cells passing through this constricted microvessel. Panels **c** and **d** show abnormal hippocampal arterioles with strictures (arrows) crossing the pyramidal cell layer of CA1. Note the severe microgliosis in this region, as cells with small nuclei are predominant in this region. Dark intravascular substance corresponds to the Brite Vu contrast agent. Insert in **d** shows higher magnification of the corresponding arteriolar stricture. Scale, 50 µm in **a**; 20 µm in **b**; 40 µm in **c** and **d**; 10 µm for inset in **d**

**Fig. 7:**
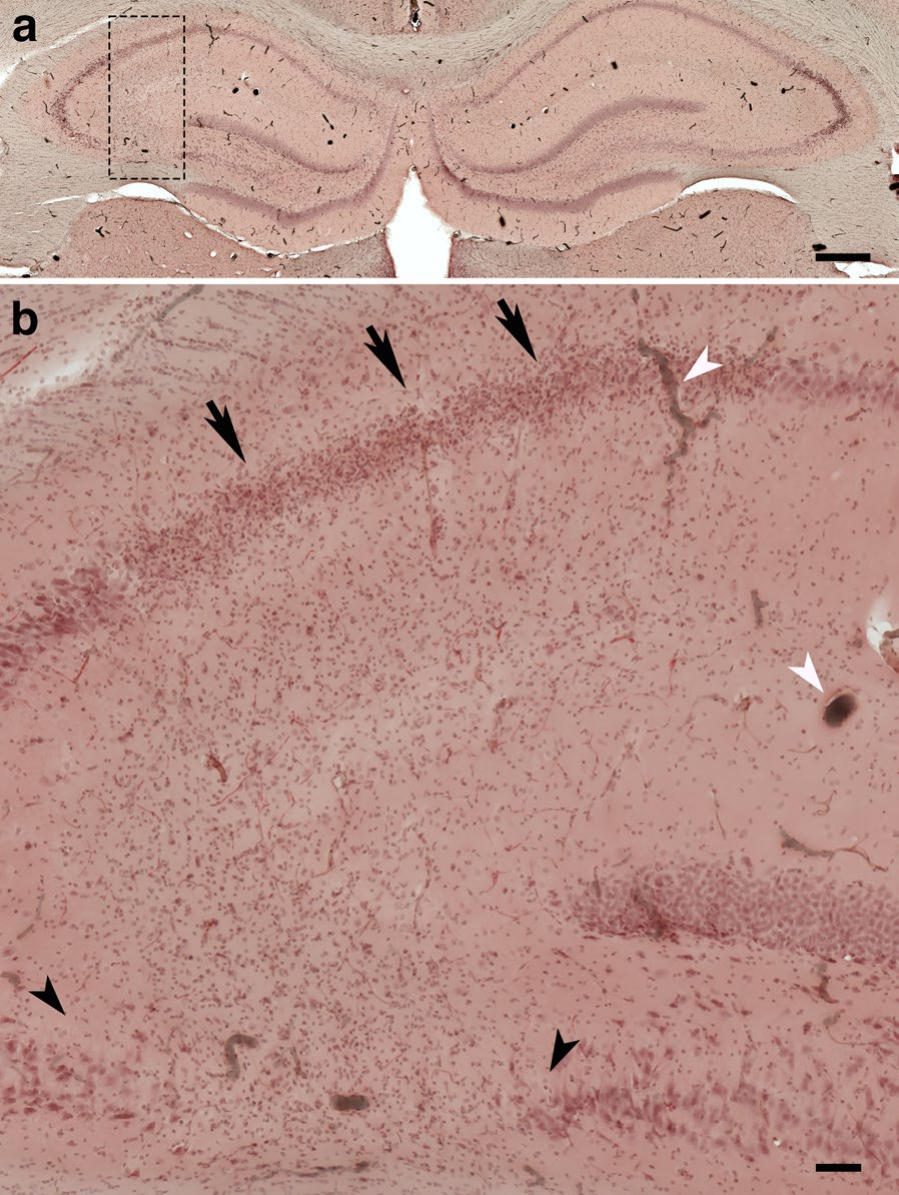
Local inflammation is associated with hippocampal neuronal loss. **a** and** b**, images of an H&E-stained coronal section of a blast-exposed brain showing massive gliosis extending locally from CA3 through the alveus (box in **a**). Notice in **b** the gliosis concentrated in the stratum pyramidale of CA1 (arrows) and the neuronal loss in the ventral CA3 associated with the inflammation (area delimited by black arrowheads). Also visible in the sections are intravascular deposits of the BriteVu micro CT contrast agent, which are indicated by white arrowheads. Scale bar, 500 µm in **a** and 100 µm in **b**

Pathological examination of the hippocampus in H&E-stained sections from this animal revealed a region of hypoperfused microvasculature in the stratum radiatum in which capillaries contained deformed elongated erythrocytes (Fig. 6a, b). In addition, abnormalities including strictures and kinks were present in some vessels crossing the strata oriens, pyramidale and radiatum (Fig. 6c, d). Associated with these vascular alterations was local neuronal loss in the hippocampal pyramidal layers CA1 and CA3 with superimposed gliosis, characterized by the infiltration of cells with small nuclei (Figs. 6c, d and 7).

**Fig. 8:**
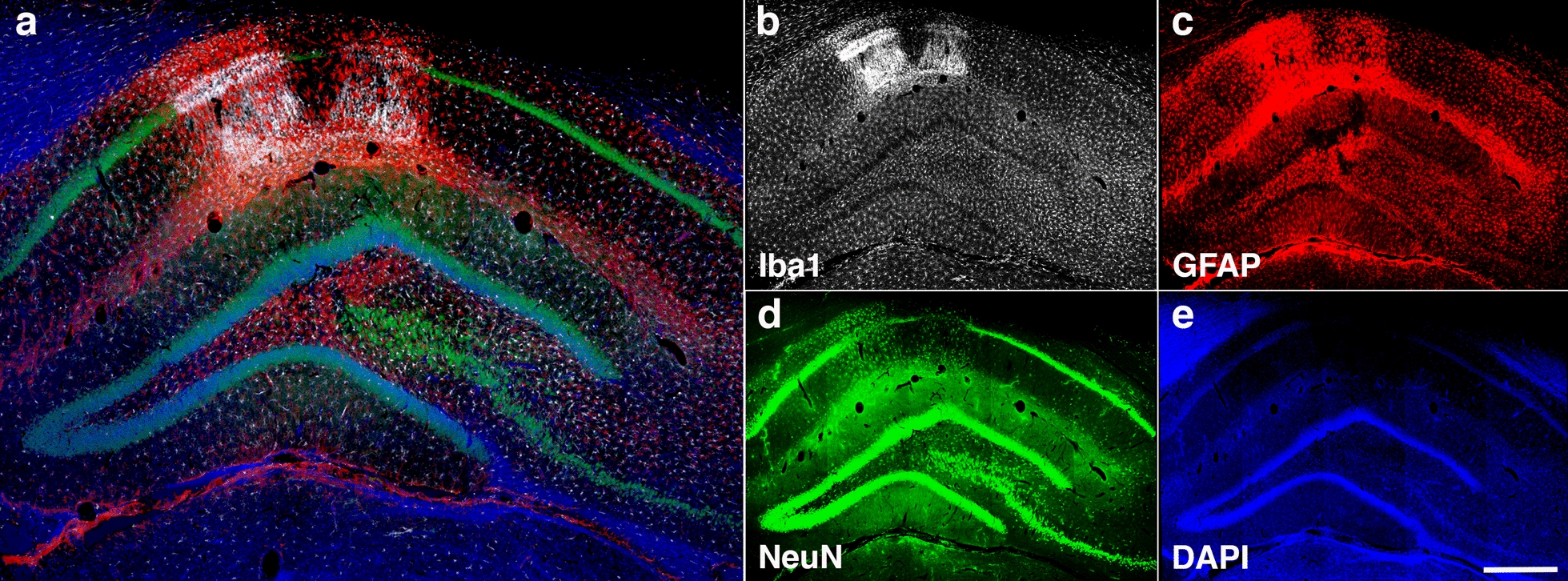
Microgliosis and astrocytosis are associated with hippocampal neuronal loss. Photomicrographs of a coronal section of the hippocampus of a blast-exposed rat with local gliosis immunostained for the identification of microglia (Iba1, white), astrocytes (GFAP, red) and neurons (NeuN, green). **a**, Merged image; **b**, Iba1 staining; **c**, GFAP staining; **d**, NeuN staining; and **e**, DAPI staining (blue). Note the loss of CA1 NeuN^+^ pyramidal cells (**d**) associated with the gliosis. Scale, 1 mm in **a** and 500 µm in **b-e**

**Fig. 9:**
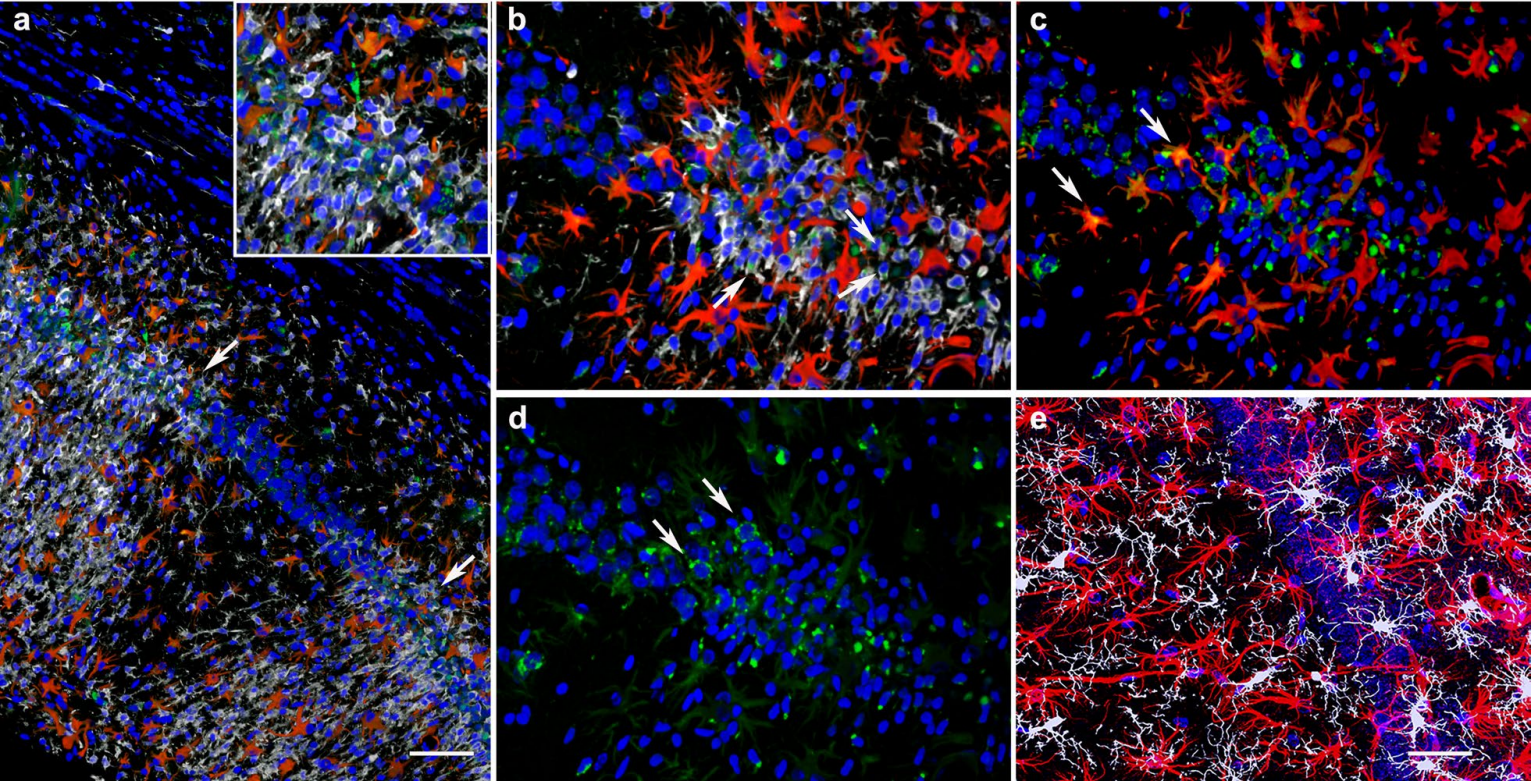
TUNEL-positive cells are associated with activated microglia and reactive astrocytes in the hippocampal CA1 region of a blast-exposed rat. Shown are images of the hippocampal CA1 region infiltrated with activated unramified ameboid microglia (Iba1, white) and reactive astrocytes (GFAP, red), and stained for TUNEL (green). **a**, Merged panoramic image. Arrows indicate affected CA1 subregions. Insert in **a** shows higher magnification of an affected CA1 subregion. Note that most of the TUNEL-positive structures (green) are associated with activated microglia (white, insert). **b**, Merged higher magnification image showing loss of large neuronal nuclei and their replacement by Iba1 positive microglial cells with small condensed nuclei (Iba1, white; arrows). **c**, TUNEL-positive astrocytes (TUNEL, green; GFAP, red; arrows). **d**, TUNEL staining. Arrows indicate TUNEL-positive neuronal nuclei in the affected CA1 subregion. **e**, Control non-blast exposed rat (Iba1, white; GFAP, red; TUNEL, green). DAPI, blue. Scale, 100 µm in **a**, 50 µm in **b-e**

**Fig. 10:**
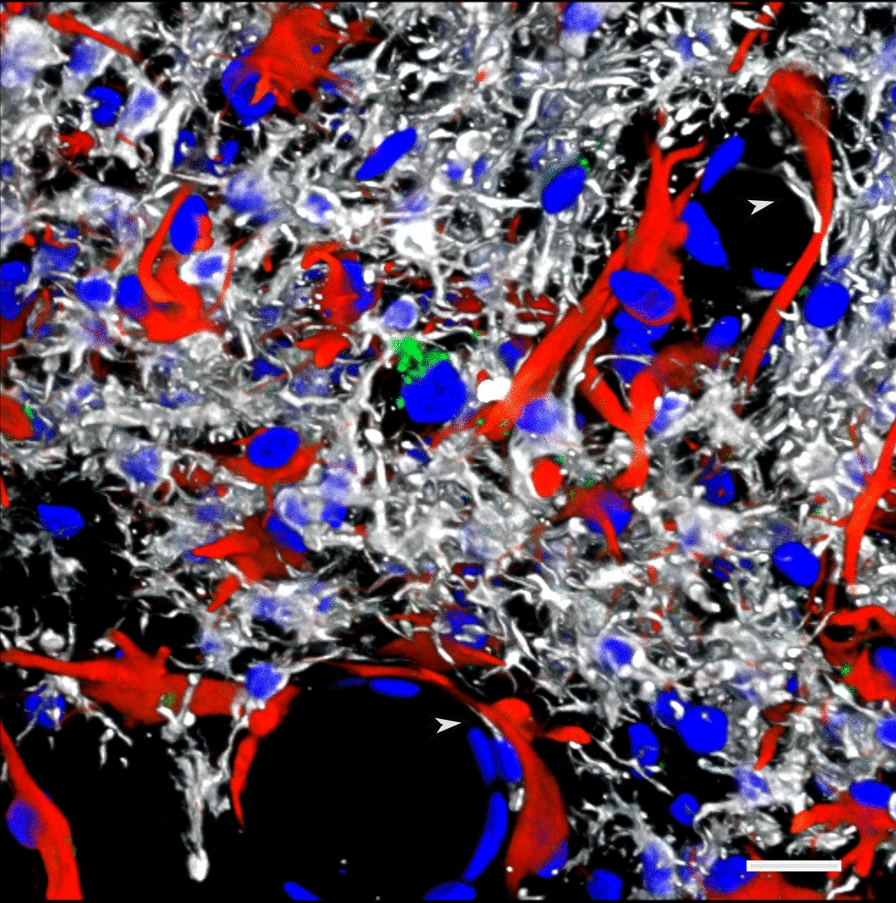
Perivascular gliosis. Severe perivascular microgliosis and reactive astrocytosis in the ventral region of the strata radiatum and lacunosum moleculare. Microglia (Iba1, white) and astrocytes (GFAP, red) with TUNEL staining (apoptosis, green) and DAPI staining (blue). Note the relatively few apoptotic bodies (green) in this region in association with microglia. Arrowheads show microglia under astrocytes lining the adventitia. Scale, 20 µm

Immunohistochemical analyses identified local infiltration of activated Iba1-expressing microglial cells and reactive GFAP^+^ astrocytes expanding from the stratum oriens through the stratum radiatum and into the stratum lacunosum moleculare (Fig. 8). Activated microglia had replaced most of the CA1 pyramidal neurons in the affected regions. Moreover, TUNEL staining identified apoptotic bodies mainly activated microglia but also reactive astrocytes and pyramidal neurons (Fig. 9). Few apoptotic bodies were observed in the severe gliosis associated with the ventral stratum radiatum and lacunosum moleculare (Fig. 10).

### Infiltrating microgliosis and reactive astrocytosis are associated with dendritic alterations and degradation of the vascular ECM

**Fig. 11:**
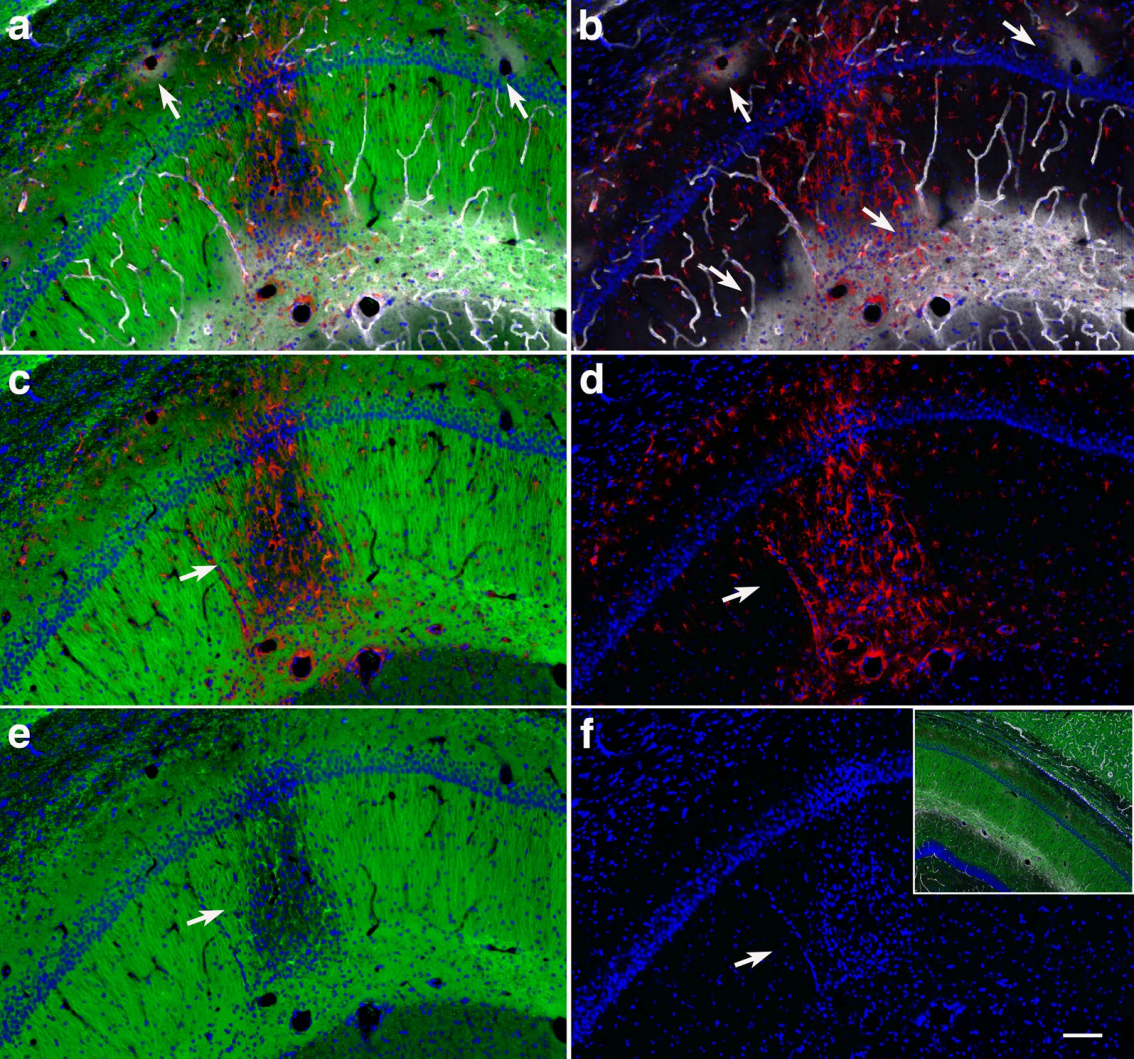
Hippocampal inflammation is associated with neuronal degeneration and degradation of vascular ECM. Shown are images of a hippocampal region in which a local gliosis that extends through the strata oriens, pyramidale, radiatum and lacunosum moleculare overlaps the dendritic degeneration in stratum radiatum. β-III tubulin (Tuj) (neuronal, green), GFAP (astrocytes, red), collagen type IV (vascular, white), and DAPI (blue) staining. **a**, merged image; **b**, reactive astrogliosis (red) overlapping a halo of perivascular collagen type IV immunostaining (white) in the lacunosum moleculare and in large vessels in the oriens (arrows); **c**, dendritic atrophy associated with inflammation (arrow); **d**, reactive astrogliosis (red); **e**, dendritic degeneration (arrow); and **f**, DAPI staining (blue). Note the small nuclei corresponding to microglia in the region showing dendritic degeneration and reactive astrogliosis. Insert in **f** shows the contralateral hippocampal region without inflammation (β-III tubulin, green; GFAP red; collagen type IV, white, and DAPI, blue). Scale, 200 µm

**Fig. 12:**
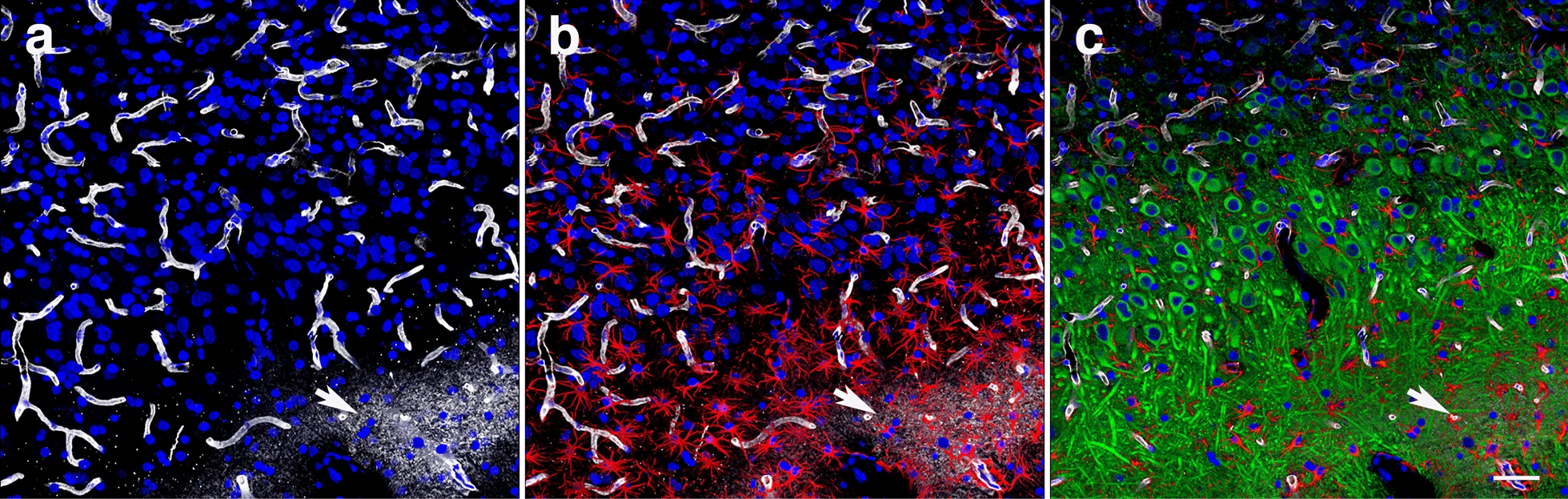
Vascular ECM degradation, gliosis and dendritic atrophy. **a**, Venular degradation of collagen type IV (white) in the stratum radiatum. Note the perivascular halo of collagen type IV- immunoreactive material (arrow). **b**, Reactive astrogliosis (GFAP, red). The arrow indicates an area of vascular degradation with respect to astrogliosis. **c**, Area within the stratum radiatum showing dendritic atrophy (arrow) overlapping area with vascular degeneration (ECM degradation). Also note collagen type IV immunostaining of the microvasculature even in the absence of antigen retrieval (pepsin treatment). DAPI staining (blue). Scale, 50 µm

**Fig. 13:**
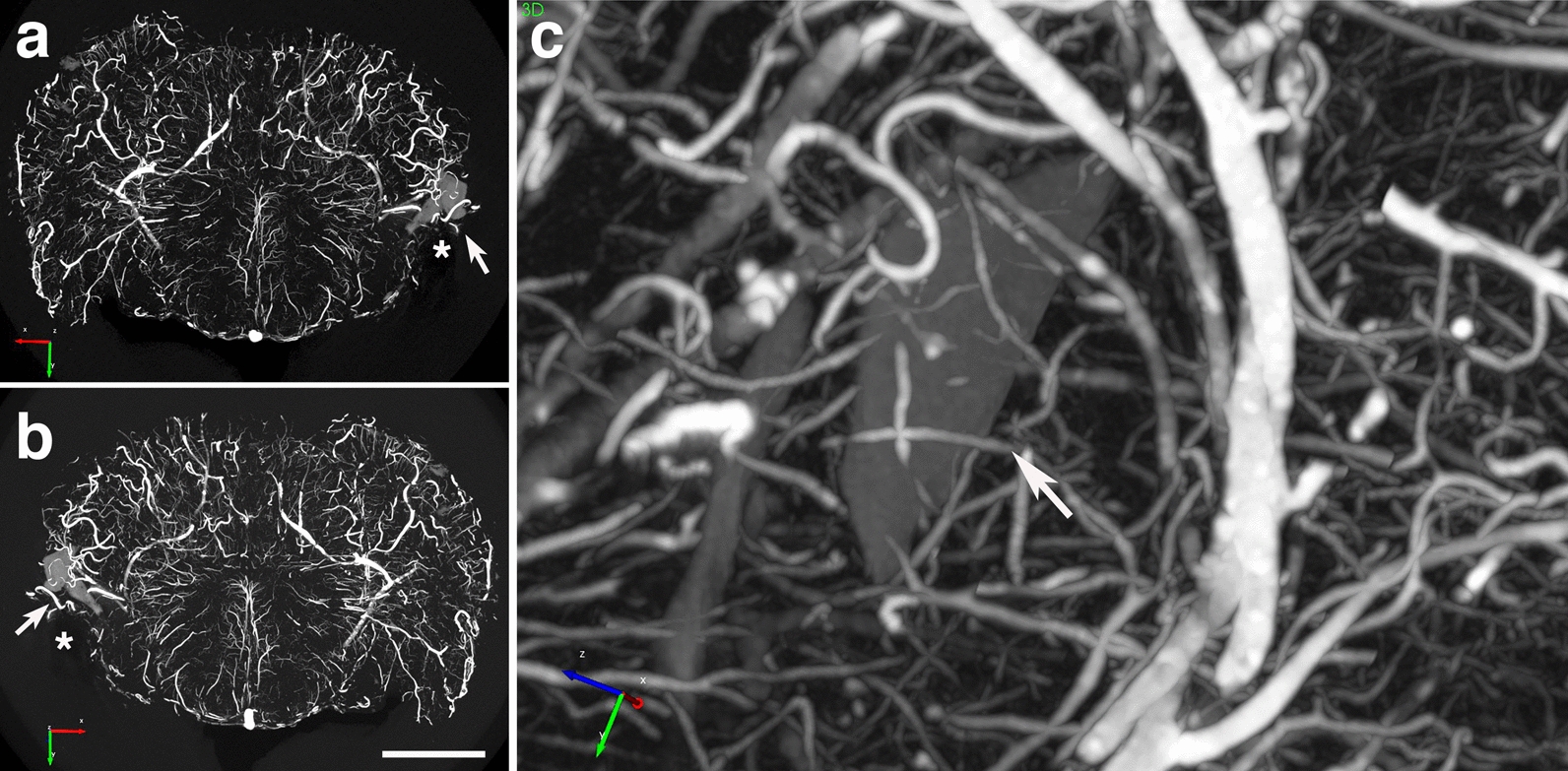
Vascular fragility. Coronal optical section of a brain micro-CT scan (approximate interaural 4.17–1.92 mm) of a blast-exposed rat perfused with Brite Vu contrast agent. **a**, Rostral and **b**, caudal views showing vascular leakage of the contrast agent into the parenchyma (arrows). Note the attenuated vasculature (not perfused) in the enthorhinal cortex below the leakage (asterisk in **a** and **b**). **c**, Higher magnification image with different orientation showing vascular leakage (arrow). Scale, 3 mm in **a** and **b**; 1.3 mm in **c**

Neuronal degeneration at the inflammation site was further investigated by immunohistochemistry with a monoclonal antibody against the neuronal marker β-III tubulin (Tuj), a major constituent of microtubules (Fig. 11). Degeneration of distal and proximal CA1 dendritic processes was observed in the strata oriens and radiatum and overlapped the reactive GFAP-immunoreactive astrogliosis and microgliosis that expanded into the stratum lacunosum moleculare (small nuclei, Fig. 11). In addition, visualization of the vasculature with antibodies against collagen type IV, a structural component of the vascular ECM, showed diffuse perivascular staining in the distal ventral stratum radiatum, in the lacunosum moleculare and in large vessels in the alveus and stratum oriens (Fig. 11a and b). Collagen type IV-positive microvasculature in the inflammatory region was absent from the stratum radiatum and all around the proximal dendrites of the CA1 neurons (Fig. 11a, b). The distribution of reactive astrogliosis and microglial infiltration paralleled the hippocampal dendritic and vascular degenerative processes (Figs. 11 and 12). Activated microglia could be observed under astrocytes lining the adventitia and even within the vascular lumen in the stratum lacunosum moleculare (Fig. 10). Vascular fragility, an indicator of the generalized cerebral vascular dysfunction in this animal, was further illustrated by the leakage of perfused contrasting agent into the brain parenchyma as observed on micro-CT scanning (Fig. 13).

### Synaptic structural proteins are downregulated in the hippocampus of blast-exposed rats

**Fig. 14:**
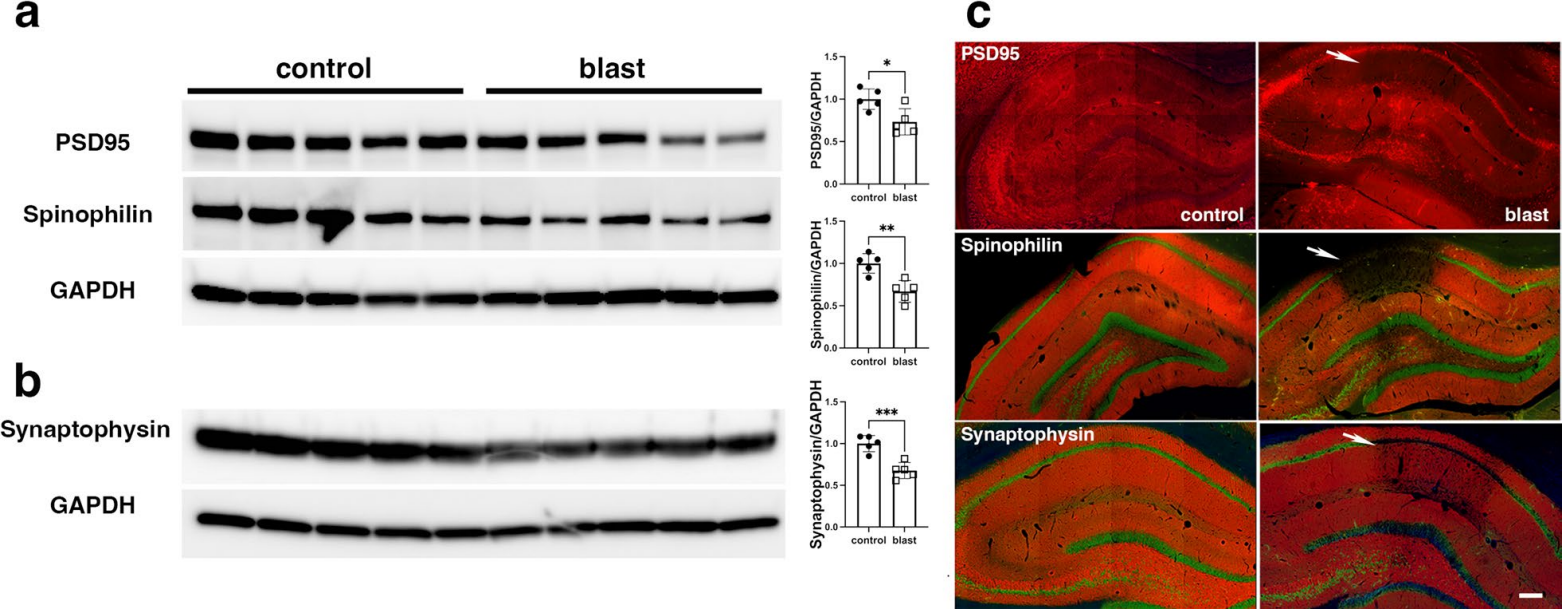
Decreased levels of synaptic structural proteins in the brains of blast-exposed rats. **a**, Hippocampal extracts from control and blast-exposed rats (n = 5/ group) derived from rats in cohort 2 [64] and sacrificed at 13 months after blast exposure were analyzed by Western blot with antibodies against the synaptic proteins PSD95 (postsynaptic), synaptophilin (postsynaptic spines) and synaptophysin (presynaptic vesicles). **a**, Blotting for PSD95 and spinophilin were performed sequentially on the same blot followed by probing for GAPDH as a loading control. **b,** Synaptophysin was probed in a separate blot followed by its own GAPDH control. Each lane in the Western blots is from an individual animal. Levels of synaptic proteins normalized to GAPDH are shown in the bar graphs (*, p < 0.005; **, p < 0.001; ****, p < 0.0001, unpaired *t*-tests). **c**, Immunohistochemical analyses of synaptic protein expression in the hippocampus of control and blast-exposed rats with specific antibodies. NeuN immunostaining (neuronal) is shown in green. Note that in regions affected by severe gliosis (arrows), levels of the synaptic structural proteins are further decreased. Scale, 200 µm

As inflammation was found to be associated with CA1 dendritic atrophy, we investigated the levels of the synaptic proteins PSD95 (postsynaptic), synaptophilin (postsynaptic spines) and synaptophysin (presynaptic vesicles) in the hippocampus of blast-exposed and control rats (13 months post-exposure). Immunoblot analysis showed a significantly decreased level of these synaptic proteins in the hippocampus of blast-exposed rats (Fig. 14a), which by immunostaining was particularly notable in the hippocampal regions experiencing inflammatory cell infiltration (Fig. 14b). As the CA1 subregion receives dual inputs from layer III of the entorhinal cortex and from CA3, our results indicate that synapses involving these neuronal communications are affected by the ongoing inflammatory processes.

### Late chronic blast-induced ultrastructural alterations

**Fig. 15:**
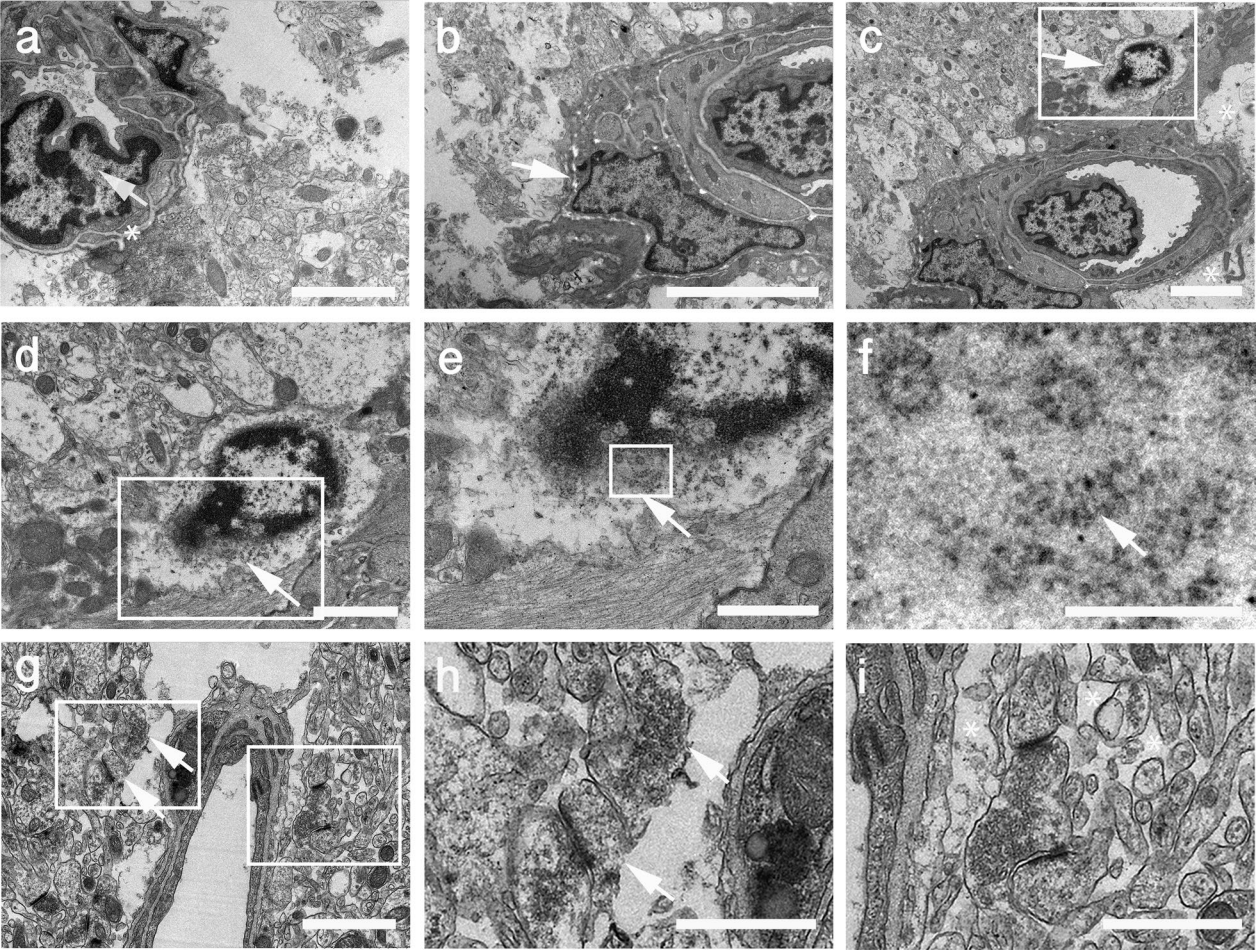
Late chronic ultrastructural alterations in the motor cortex of a blast-exposed rat. **a**, Arteriolar spasm with partially occluded lumen next to a tear. Endothelial cell with an enlarged nucleus and irregular electron density within the ECM (asterisk) indicative of vascular remodeling and cellular detachment [74], (arrow). Note the degenerating neuropil next to the tear. **b-f**, Arteriole with irregular discontinuous ECM (arrow in **b**), attached swollen astrocytic feet (asterisks in **c**) and apoptotic microglia (arrow in **c**). Boxed region (**c**) shown at higher magnification in (**d**). **d**, Apoptotic microglia enmeshed in disrupted cytoplasm immediately adjacent to affected vasculature. Note swollen mitochondria along bottom left. **d-f**, Lack of nuclear membrane in the apoptotic microglia with folded chromatin fibers present in the cytoplasm (arrows **d-f**). **g-i**, Enlarged paravascular space, perivascular astrocytic degeneration and empty spaces in the neuropil. Boxed regions are shown at higher magnification (**h, i**). Arrows in **g** and **h** denote membrane damage in a postsynaptic spine and in a presynaptic axonal terminal. Asterisks in **i** indicate empty spaces within the neuropil. Note intact multisynaptic bouton in perivascular region immediately adjacent to a blast damaged vessel. Scale, 2 µm in **a**,** b**,** d**,** g**; 4 µm in **c**; 1.2 µm in **e;** 600 nm in** f** and 1 µm in **h** and **i**

Electron microscopy analyses in the vicinity of a blast-induced tear in the motor cortex confirmed that late vascular alterations in the brain of blast-exposed animals are associated with degenerative processes in the neighboring neuropil (Fig. 15). Vascular alterations included swollen endothelial nuclei, partially occluded lumen and discontinuous laminae (Fig. 15a–c), the latter being indicative of ECM remodeling. Contiguous to a vessel undergoing ECM remodeling, we also identified a small apoptotic microglial cell with a small hollow nucleus and unfolded chromatin released into the cytoplasm (Fig. 15c–f). Chromatin degradation may be responsible in part for the TUNEL reactivity observed in activated microglia associated with inflammation (Fig. 9). Cell membrane alterations were also detected in presynaptic axonal terminals and neighboring dendritic spines in regions adjacent to enlarged paravascular spaces with astrocytic loss, (Fig. 15g–i). Other alterations in the neighboring neuropil included cellular debris within the enlarged paravascular spaces whose ultrastructural origin could not be identified.

### Vascular abnormalities are associated with shear-related lesions

**Fig. 16:**
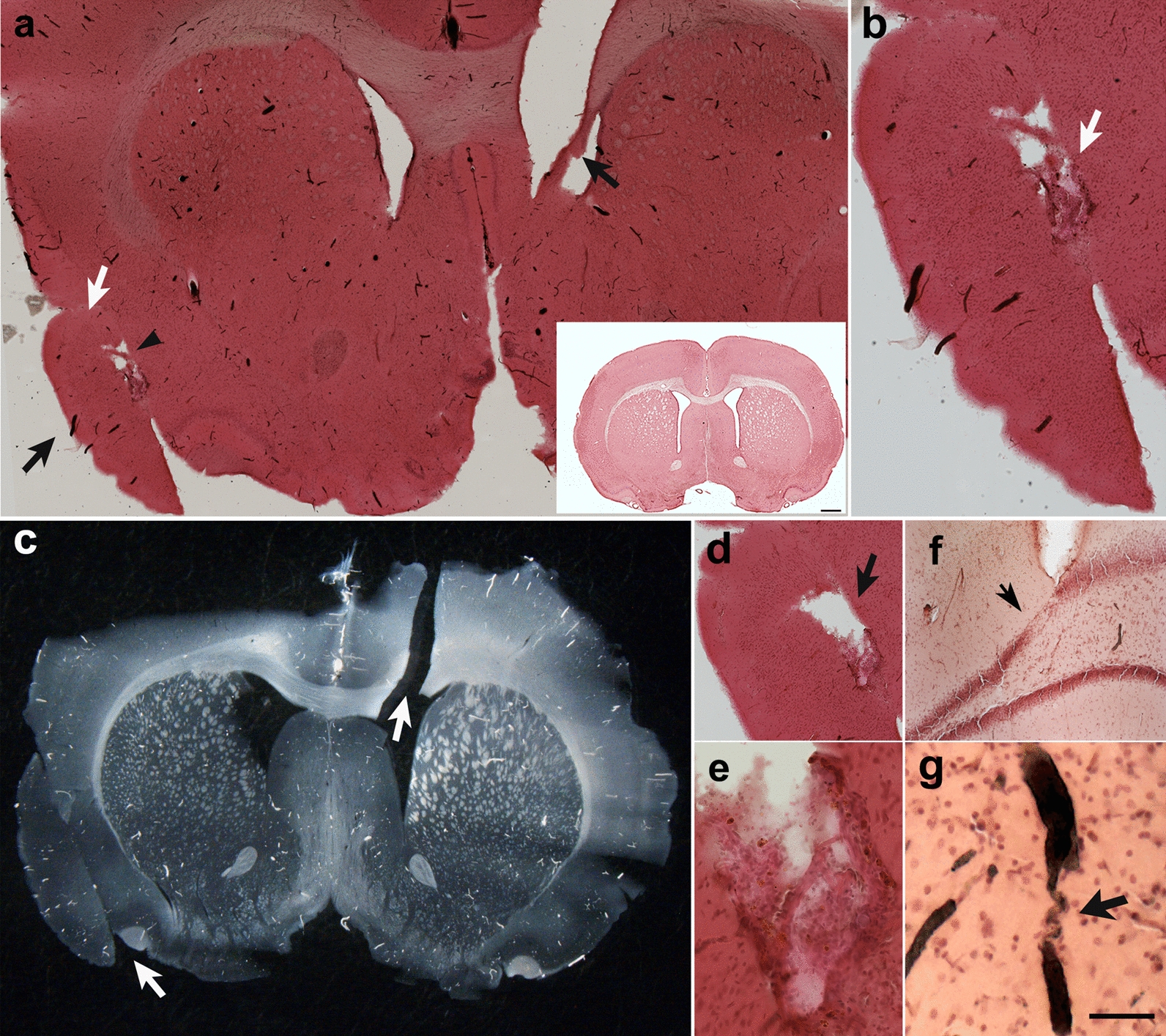
Blast-induced tears, tissue repositioning and arteriovenous malformations in the brain of a blast-exposed rat. **a**, H&E-stained coronal section of the brain of a blast-exposed rat (13 months post-exposure) showing ventrally repositioned tissue including part of the insular cortex (black arrow). The contralateral hemisphere shows tears expanding through the corpus callosum and lateral ventricle (black arrows). The white arrow denotes a region of tissue reattachment. Note the cavity next to the displaced insular tissue with large vessels (arrowhead). Insert in **a** shows the normal morphology in a section from a non-blasted control rat stained with H&E. **b**, Higher magnification of **a**, showing the location where displaced tissue reattachment occurred and arteriovenous malformations in the cavity (arrow). **c**, Diascopic dark-field image of a neighboring coronal section showing the repositioned insular cortical tissue and a tear expanding from the lateral ventricle across the corpus callosum and motor cortex (arrows). Other tears were present that affected the integrity of the tissue section. **d**, Cavity generated from torn/repositioned tissue with arteriovenous malformations in another neighboring section. **e**, Higher magnification image of arteriovenous malformations. **f**, Neuronal loss in the upper blade of the dentate gyrus next to a tissue tear. **g**, Tortuosity in a hippocampal artery (arrow). Dark intravascular substance corresponds to the Brite Vu contrast agent used to perfuse this animal for the micro-CT analysis. Scale, 1 mm in **a**, 0.5 mm in **b**, 0.7 mm in **c**, 0.5 mm in **d**, 100 µm in **e**, 0.6 mm in **f**, 200 µm in **g**

We previously reported a type of brain shear lesion, unique to blast-exposed animals that appears to follow the fault line of penetrating cortical vessels [28]. Such lesions contain at their margins a glial reaction and evidence of neuronal injury indicating their chronicity and non-artifactual nature [28]. Microscopic examination of H&E-stained coronal sections from blast-exposed rats identified a blast-induced shear injury that expanded through the insular cortex (Fig. 16a–d). This lesion involved shearing and repositioning of the cortical insular tissue. Layers I, II and III of the granular, dysgranular and agranular insular cortex were torn, bent, perpendicularly repositioned and reattached to the deeper layers.

**Fig. 17:**
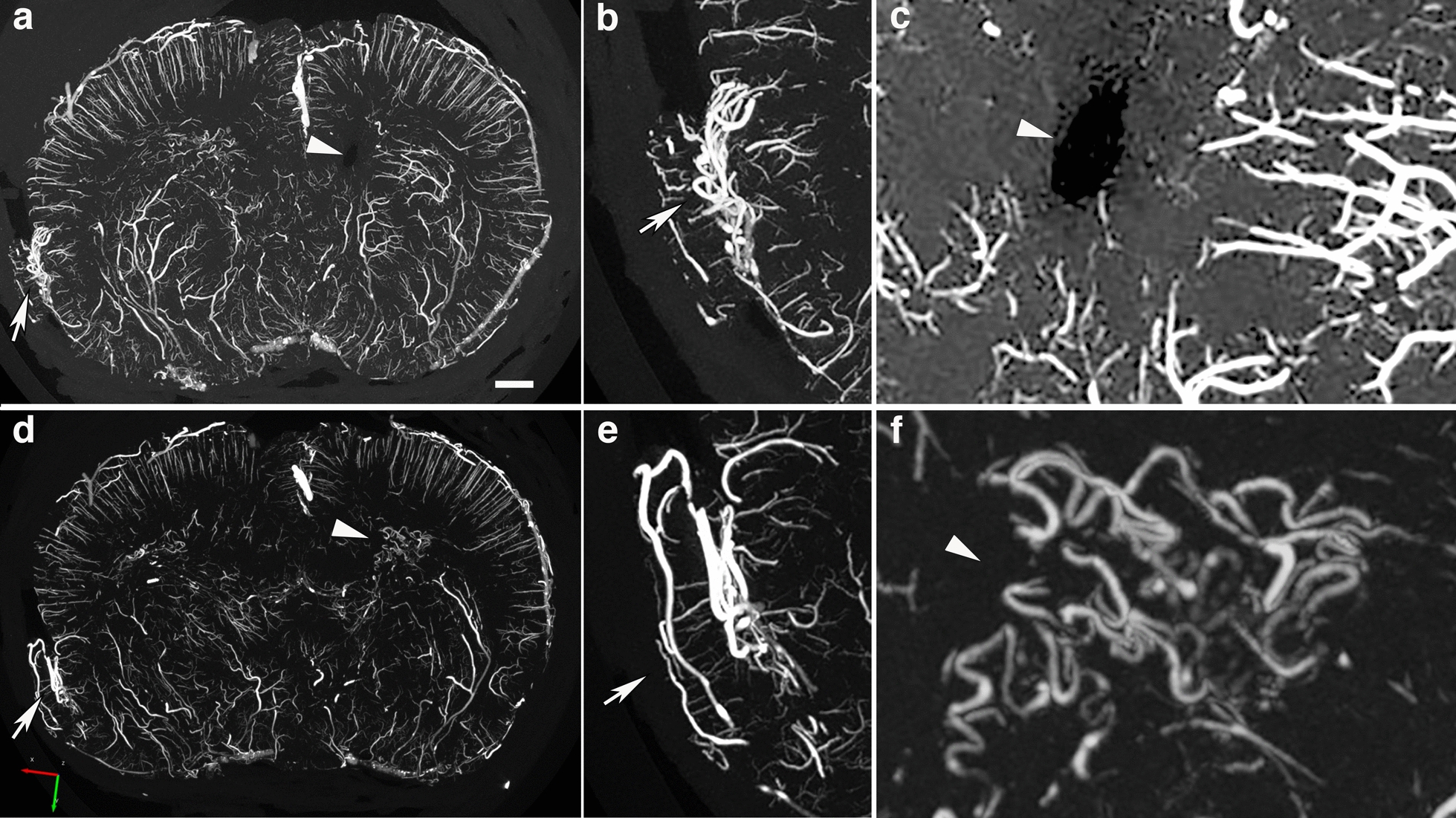
X-ray micro-CT scan showing blast-induced arteriovenous malformations. **a-f**, two serial optical coronal sections (1.5 mm-thick) of a BriteVu-perfused brain are shown, along with additional sections in Figs. 15 and 16. **a-c**, Interaural 9.84–8.34 mm; **d-f**, interaural 8.34–6.84 mm. Arrows in **a-b** and **d-e** denote the formation of arteriovenous malformations within the tear-damaged insular cortex. Arrowheads in **a** and **c** indicate a small area with much lower X-ray attenuation than the surrounding tissue, consistent with an air void within the tissue when compared to the surrounding areas. This region maps to the damaged lateral ventricle with a tear extending through the corpus callosum and into the motor cortex (Fig. 15). Arrowheads in **d** and **f** indicate an artery with high tortuosity in the rostral hippocampus. Scale, 1 mm in **a** and **d**; 2.2 mm in **b** and **e**; 0.3 mm in **c** and **f**

At the core of this lesion was an abnormal vasculature that, when visualized through the micro-CT-reconstructed angioarchitecture, appeared as an entangled, tortuous and convoluted vascular nidus resembling an arteriovenous malformation (Figs. 16d and 17a, b). The contralateral side contained a shear lesion that expanded dorsally through the lateral septal area next to the 3^rd^ ventricle and rostral hippocampus, corpus callosum and into the motor cortex. The upper blade of the dentate gyrus next to the tear showed neuronal loss (Fig. 16f). Vascular alterations including arterial constriction and tortuosity were observed in the vicinity of this lesion (Fig. 16g). A micro-CT optical section (interaural 9.84–8.34 mm) revealed the presence of an abnormal vascular nidus malformation next to the lateral ventricle and hippocampus that also resembled an arteriovenous malformation (Fig. 17c).

**Fig. 18:**
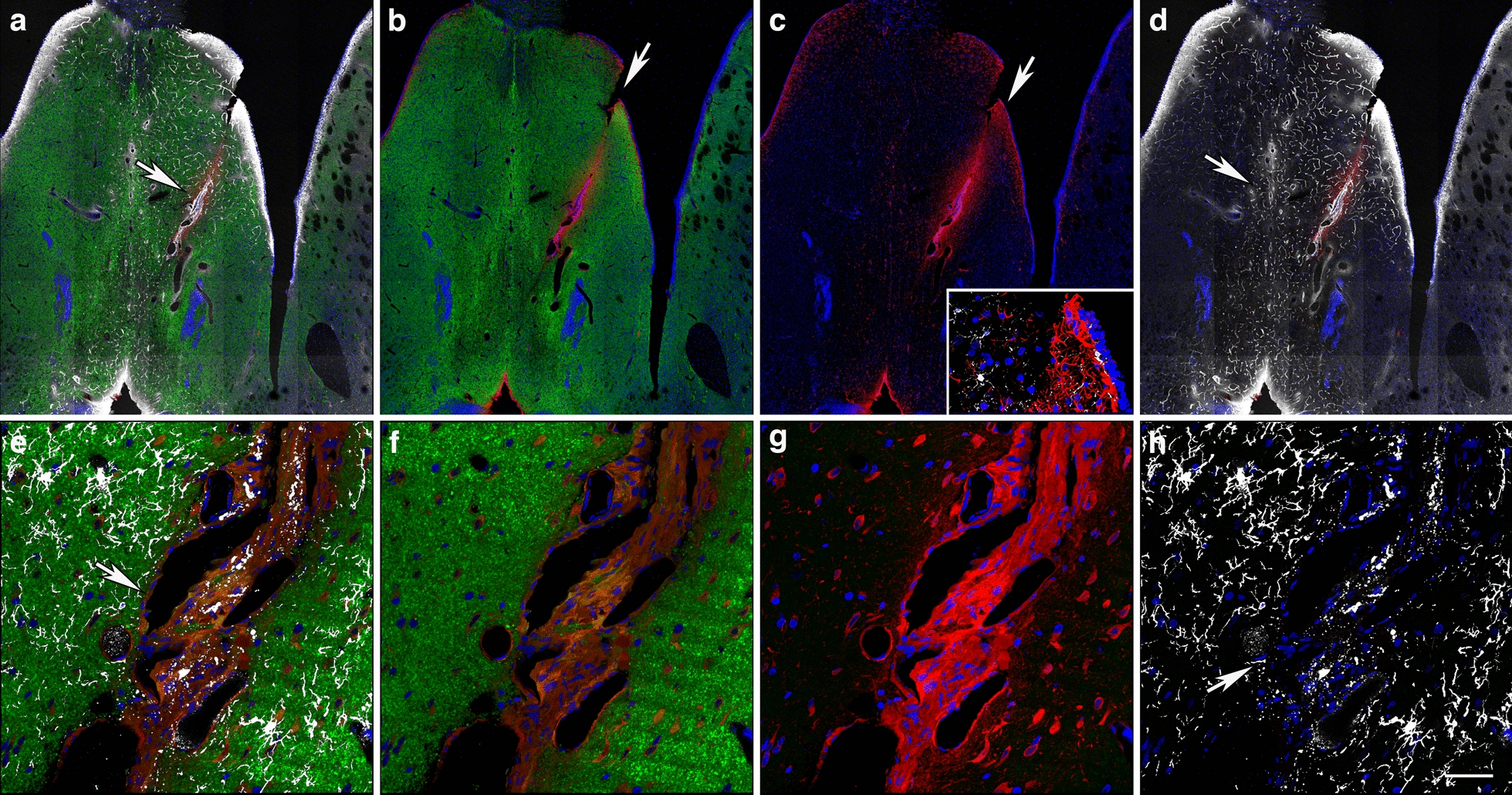
Blast-induced tear, tissue repositioning and vascular remodeling. **a-d**, Section stained with antibodies against β-III tubulin (neuronal, green), GFAP (astrocytic, red) and collagen type IV (vascular ECM, white). **a**, Merged image; **b**, β-III tubulin and GFAP immunostaining; **c**, GFAP immunostaining; and **d**; collagen type IV and GFAP immunostaining. Nuclei were stained with DAPI (blue). Note the tear (indicated by the arrows in **a**-**e** and **h**) in the lateral septum that follows a large penetrating vessel, and reactive astrocytosis associated with resulting tissue repositioning (insert in **c**) and collagen type IV immunostaining of the microvasculature in the area associated with the lesion in absence of protease pretreatment (vascular remodeling). **e–h**, Remnants of perivascular inflammation in the region of the tear illustrated above. β-III tubulin (neuronal, green), GFAP (astrocytic, red) and Iba1 (microglia, white) immunostaining. **e**, Merged image; **f**, β-III tubulin and GFAP immunostaining; **g**, GFAP immunostaining; and **h**, Iba1 immunostaining. Note in the adjacent neuropil the presence of ramified type 1 Iba1-expressing cells and the lack of ameboid microglia, while on the large vasculature only remnants of Iba1-immunoreactive material are present. Scale, 500 µm in **a-d** and 100 µm in **e–h**

**Fig. 19:**
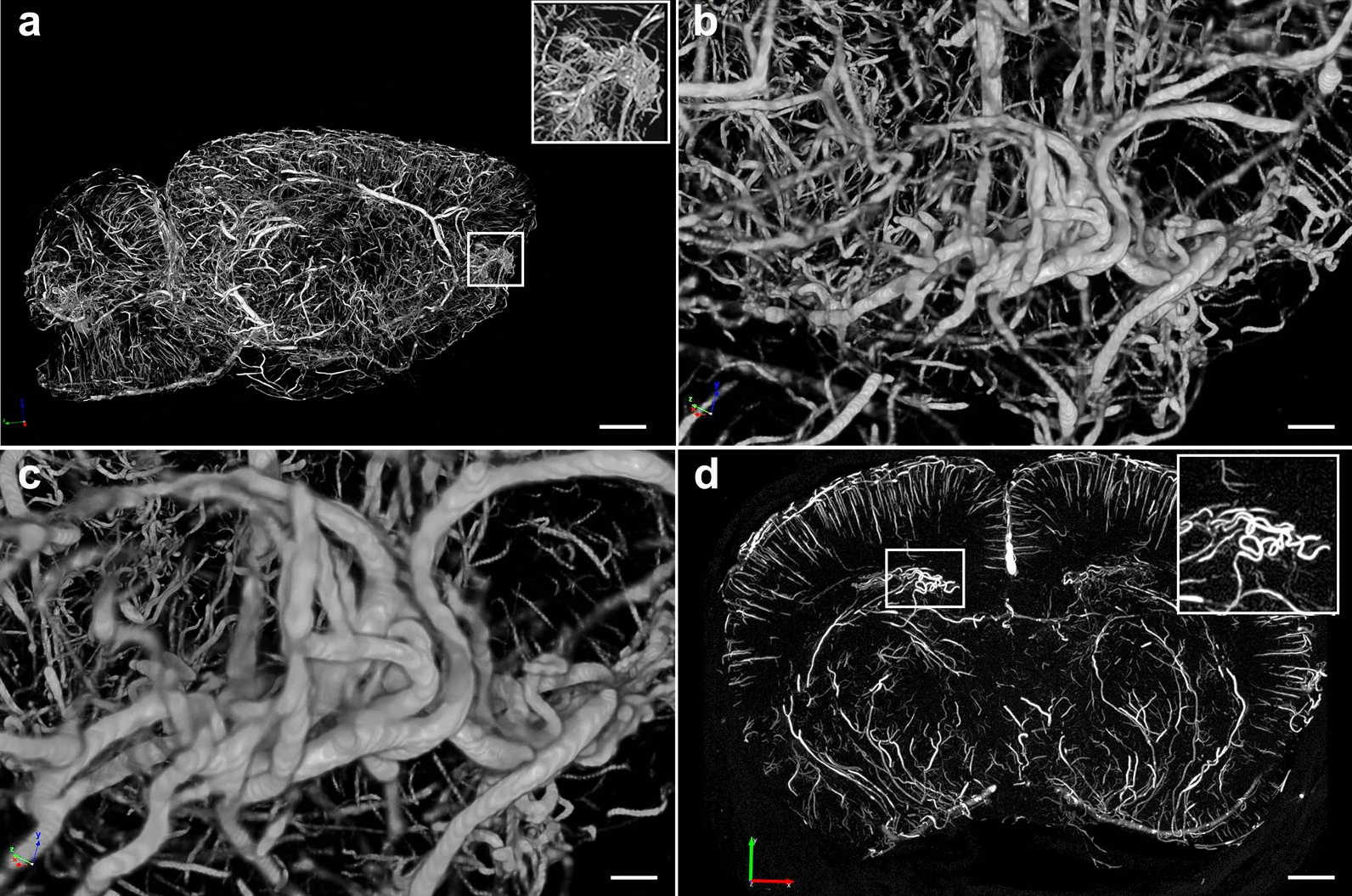
Insular arteriovenous malformations and hippocampal vascular tortuosity in a blast-exposed rat. Lateral views of the micro-CT angiogram 3D reconstruction demonstrating the intracortical arterial feeder vessels forming a large arteriovenous nidus. **a**, Lateral view of the brain vasculature. The box denotes an arteriovenous malformation in the insular cortex. Higher magnification images of this structure are seen in the insert white box in **a,** in **b** and **c**. **d**, Coronal optical section (approximate interaural coordinates 7.20–4.95 mm) showing increased arterial tortuosity in the frontal hippocampal region of one hemisphere. Scale bar, 1 mm in **a**, 0.1 mm in **b**, 50 mm in **c**, 1 mm in **d**

The micro-CT scan also revealed the presence of an opaque area within the lateral ventricle with lower X-ray attenuation than the surrounding tissue, which would be consistent with an air void or, although unlikely, soft tissue lacking hydration (Fig. 17d). A lateral shear that affected the internal wall of the 3^rd^ ventricle was also observed (Figs. 16a and 18). This lesion extending through a complex vascular array resulted in tissue repositioning around the lateral septal nuclei (Fig. 18). Moreover, around the lesion, the vasculature was stained with antibodies against collagen type IV even in the absence of protease pretreatment, indicating degradation or remodeling of the vascular ECM (Fig. 18a–d). Reactive astrogliosis in the lateral septal region was detected mainly on one side of the lesion, which indicates the direction of the shearing force. Overall, microglia in the neighboring neuropil consisted mostly of ramified Iba1^+^ cells indicative of their quiescence. However, Iba1-immunoreactive debris were associated with the abnormal vasculature, suggesting prior perivascular gliosis. Increased hippocampal arterial tortuosity was also apparent in this animal (Fig. 19).

Vascular malformations were identified in 2 of 4 blast-exposed rats studied at 13 months while none of the 5 controls studied at 13 months showed these lesions. On a review of all the micro-CT scans in Gama Sosa et al. [31], a vascular malformation was found in 1 of 6 blast-exposed rats studied at six weeks. No vascular malformations were found in 6 blast-exposed rats studied at 48 h and none were found in 17 control animals examined at 48 h (n = 6), 6 weeks (n = 6) or 13 months (n = 5). Thus, development of vascular malformations may be a delayed effect of blast exposure.

## Discussion

### Late-onset local inflammation is associated with vascular degeneration

We have previously reported the development of vascular alterations associated with enlarged paravascular spaces, perivascular astrocytic degeneration and perivascular inflammation in blast-exposed animals [31]. The present study includes further pathological analyses in these animals that confirm the presence of activated pro-apoptotic perivascular microglia associated with perivascular astrocytes. Moreover, we identified the development of regional neuroinflammation in a hypoperfused region of the hippocampus that was associated with neuronal loss in the local CA1 and CA3 regions. Vascular alterations in this area included constrictions in arterial vessels and hypoperfusion of capillaries as shown by the presence of elongated red blood cells in attenuated capillaries.

Neuroinflammation in the affected hippocampus expanded from the stratum oriens through the stratum radiatum and into the stratum lacunosum moleculare. Hippocampal blood is supplied by branches of the posterior cerebral artery, the anterior choroidal artery, and the arteries and arterioles arising from both of these vessels [17, 35, 89]. Branches of the longitudinal hippocampal artery vascularize the subregions of Ammon’s horn (CA1, CA2 and CA3) [17]. Therefore, blast-induced alterations of the hippocampal vasculature would result in regional hypoperfusion and anoxia. It is well known that CA1 neurons are particularly susceptible to anoxia and drug-induced vasospasm [16, 60, 82].

### Neuroinflammation is associated with dendritic degeneration

The CA1 subregion receives dual inputs from CA3 and from layer III of the entorhinal cortex through the stratum lacunosum moleculare. Moreover, recent viral retrograde tracing has shown non-canonical synaptic inputs to the dorsal hippocampal CA3 from ventral CA1 [50]. In the present study, immunohistochemical staining for the dendrite marker β-III tubulin in the hippocampal regions affected with neuroinflammation showed dendritic loss mainly in the stratum radiatum but not in the lacunosum moleculare. In the affected CA1 region, TUNEL-positive pro-apoptotic neurons, reactive astrocytes and activated microglia were identified in association with dendritic degeneration. Interestingly, EM analyses identified apoptotic perivascular microglia with hollow nuclei and degrading chromatin in an affected cortical area. Similar cells could be responsible for the microglial TUNEL reactivity in regions affected by inflammation. Apoptosis of microglia may be a fundamental self-regulatory mechanism devised to limit bystander killing of vulnerable neurons. The presence of the hollow nucleus in apoptotic microglia recalls the microglial Lochkern-like cells previously described [31]. As expected, immunohistochemical analyses of the presynaptic vesicle protein synaptophysin and postsynaptic proteins PSD95 and spinophilin showed decreased levels of these synaptic components that overlapped with the activated microglial inflammation and reactive astrocytes. Lower levels of these synaptic proteins in total hippocampal extracts of blast-exposed animals were further confirmed by Western blotting, suggesting that a generalized inflammatory process may play a role in these synaptic alterations.

Emerging evidence suggests that inflammatory cells in the brain, such as microglia and astrocytes, are crucially involved in regulating synaptic structure and function [56, 91, 92]. Microglia act as far-reaching potent regulators of the extended neuron-glia network by secreting soluble mediators and by establishing direct contacts with the synaptic compartment [5]. Neuroinflammation is associated with synaptic dysfunction and is known to occur secondary to cerebral hypoxia/ischemia. Activated microglia lead to an up-regulation of pro-inflammatory cytokines, such as interleukin (IL)-1β, tumor necrosis factor (TNF)-α, IL-6, prostaglandins, nitric oxide (NO) and glutamate, causing functional and structural abnormalities of the synaptic compartment [5, 11, 20].

In reactive astrogliosis resulting from brain insults or neurodegenerative conditions, astrocytes respond by secreting extracellular effector molecules in a finely graduated continuum of progressive alterations in gene expression and cellular changes, which may be triggered or regulated by various intercellular signaling molecules, including IL-1, IL-6, IL-10, TNF-α, interferon (IFN)-γ, ciliary neurotrophic factor (CNTF), leukemia inhibitory factor (LIF), oncostatin M, fibroblast growth factor (FGF)-2, FGF-8, transforming growth factor (TGF)-α, TGF-β, amyloid beta (Aβ), lipopolysaccharide (LPS), ATP, reactive oxygen species (ROS), noradrenalin and glutamate [34, 44, 49]. In turn, activated astrocytes may secrete NO, many cytokines and chemokines, such as IL-1β, IL-6, TNF-α, CXC motif chemokine ligand 1 (CXCL1), IL-8 (CXCL8), IFN-γ–induced protein (IP)-10/CXCL10, monocyte chemoattractant protein (MCP)-1/CCL2, macrophage inflammatory protein (MIP)-1α/CCL3, macrophage migration inhibitory factor (MIF), granulocyte colony-stimulating factor (G-CSF) and granulocyte–macrophage colony-stimulating factor (GM-CSF), causing the infiltration of microglia and circulating leukocytes into the brain, leading to chronic inflammatory processes. Reactive astrocytes may also produce cytotoxins (such as Lcn2) and neurotrophic factors (brain-derived neurotrophic factor [BDNF], vascular endothelial growth factor [VEGF] and basic FGF [bFGF]). As a result, activated astrocytes play either a neurotoxic role (A1 astrocytes induced by inflammatory stimuli) promoting inflammation through upregulation of classical complement cascade genes, tissue damage and synaptic degeneration or a neuroprotective function (A2 astrocytes induced by ischemia) through trophic factors that promote either survival and growth of neurons or synaptic repair [34, 44, 49, 78].

Synaptic alterations are part of the early pathophysiology of inflammatory and degenerative disorders such as multiple sclerosis and Alzheimer’s disease [42, 88]. In Alzheimer’s disease, synapse loss correlates with cognitive decline and is present after the initial symptomatic manifestation of the disease [73, 81]. Growing evidence indicates that TBI leads to significant dendritic and synaptic degeneration [42]. In Alzheimer’s disease, synapses appear to play a role in disease progression through the trans-synaptic spread of pathological tau [77]. This further raises the question of whether synapses play a role in the progression of neurodegeneration following TBI [42].

### Neuroinflammation overlaps alterations in the vascular ECM

Collagen type IV is a major constituent of the vascular basement membrane and forms an extensively cross-linked oligomeric network organized into a sheet-like network through covalent binding between individual collagen IV triplexes. Collagen type IV protomers are normally covalently linked together through sulfilimine bonds [86], but additional protein cross-linking takes place over time by advanced glycation end-products [7, 61, 84], which interferes with immunodetection [48]. It is well established that immunological detection of collagen type IV in normal adult rodents (but not in embryos and young animals) requires pretreatment with a protease (i.e., pepsin) to expose the antibody-recognized epitopes [26]. We have previously established that vascular collagen type IV around blast lesions can be immunodetected in the absence of protease pretreatment most likely due to the presence of proteases that are involved in ECM remodeling [24, 27]. We also determined in later post-blast observations that some of the blast-affected arterioles may present deficits of adventitial collagen type IV with the smooth muscle layer of the media being exposed [31].

In the present study, collagen type IV immunostaining without pepsin pretreatment labeled the hippocampal vasculature in a blast-exposed animal. In the large vessels of the stratum lacunosum moleculare and stratum oriens, the perivascular collagen type IV staining was diffuse, indicating that collagen type IV-immunoreactive material was scattered around the vessels. This implies that degradation products of the vascular collagen type IV were scattered in the tissue. Moreover, DAPI staining and immunohistochemical analysis of a neighboring section showed correspondence between neuroinflammation (gliosis) and degradation of vascular collagen type IV.

Under pathological conditions, vascular matrix proteins undergoing proteolytic processing yield bioactive fragments that influence vascular ECM remodeling [90]. Endogenously produced non-collagenous domain (NCl) fragments of human α1 (IV), α2 (IV) and α3 (IV) have been identified as anti-angiogenic, corresponding to 26-kDa arrestin, 24-kDa canstatin, and 28-kDa tumstatin [15, 67]. When the α2 (IV) NCl domain of collagen type IV was added to bovine retinal microvascular endothelial cells, it inhibited endothelial cell early attachment, proliferation, and in vitro angiogenesis. It also induced endothelial cell apoptosis and inhibited angiogenesis in an oxygen-induced retinopathy model [14]. Pentastatin, a 20-amino acid peptide from the α5 fibril of collagen type IV, suppressed vessel growth in an in vitro assay of angiogenesis and in an in vivo tumor model [90].

Destruction of vascular matrix proteins leads to vascular cell or blood-borne leukocyte accumulation, proliferation, and neointima formation within the vascular wall, blood vessels prone to uncontrolled enlargement during blood flow diastole, tortuous vein development, and neovascularization from existing pathological tissue microvessels [90]. In addition, collagen degradation fragments released from ECM by metalloproteases may propagate vascular smooth muscle apoptosis by calpain-mediated inactivation of cross-linked inhibitor-of-apoptosis protein (xIAP) [87]. Electron microscopy confirmed areas of ECM remodeling in blast-affected regions.

### Chronic synaptic alterations occur in the brains of blast-exposed rats

Alterations in neuronal physiology, mainly synaptic function, are expected to be at the core of the chronic blast-induced behavioral alterations. Biochemical analyses in the present study showed decreased levels of synaptic proteins (PSD95, synaptophysin and spinophilin) in total hippocampal extracts of blast-exposed rats (13 months post-exposure). This phenomenon was exacerbated in areas with severe microgliosis and reactive astrocytosis. Synapse alterations were also found in areas with enlarged paravascular spaces and microglial apoptosis that included astrocytic and vascular degeneration.

Perivascular astrocytic atrophy and degeneration are early (acute) and late (chronic) hallmarks of blast-exposure and are associated with vascular alterations. In our model, astrocytic alterations occur before any blast-induced behavior alterations and neuroinflammation can be detected. Late in the disease progression, activated TUNEL-positive microglia are commonly found with perivascular astrocytes on large vessels in blast-exposed animals. Perivascular astrocytic atrophy and degeneration should lead to a reduction in the astroglial coverage of blood vessels and synapses, contributing to dysfunction in the neurovascular unit and tripartite synapse.

**Fig. 20:**
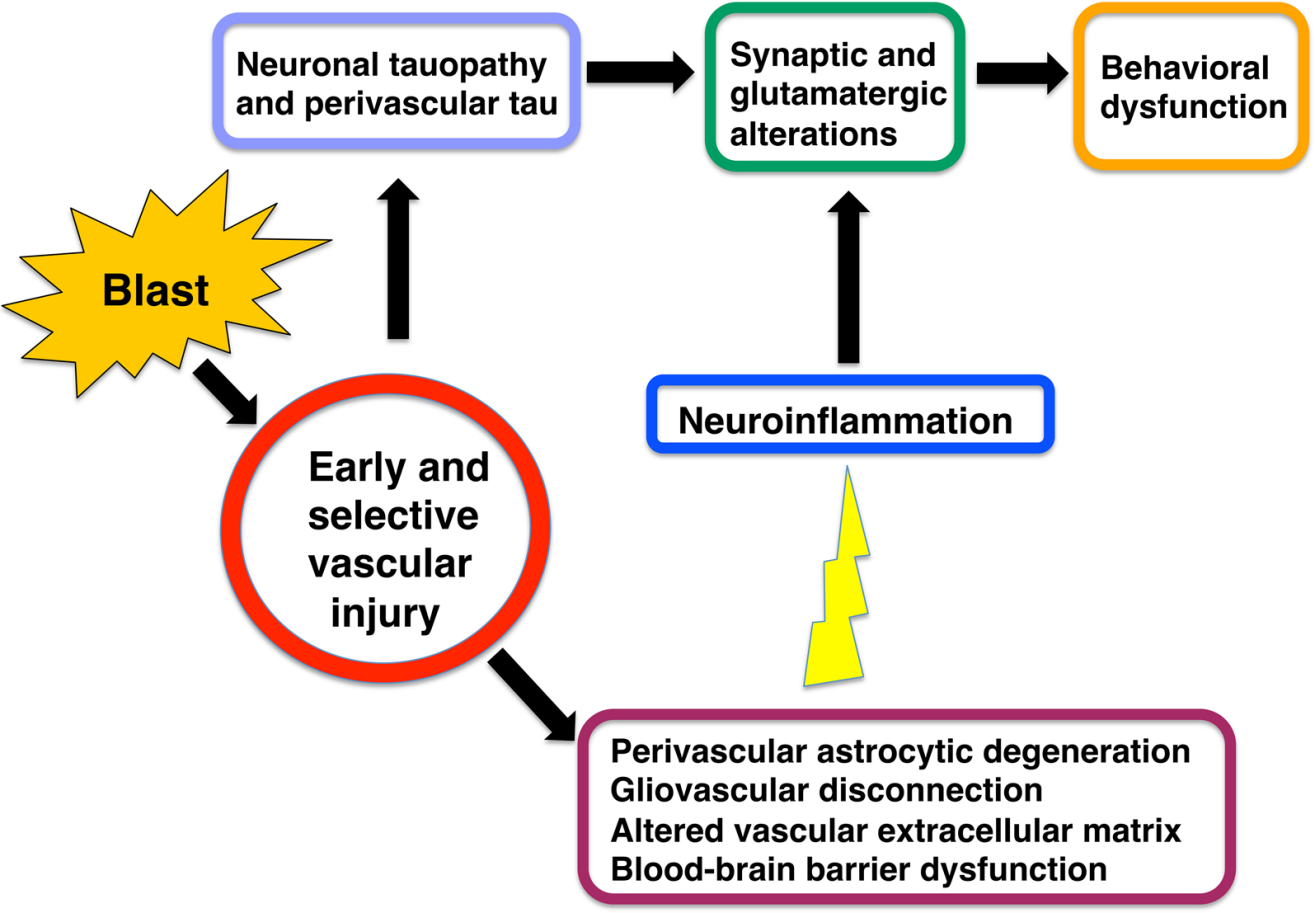
Hypothetical scheme for how early vascular injury leads to a delayed and chronic neurobehavioral phenotype. Blast exposure is associated with an early and selective vascular injury [27] associated with damage to and loss of perivascular astrocytes [29] with altered vascular extracellular matrix proteins ([27, 29, this paper], leading to presumed blood brain barrier dysfunction. Perivascular and neuronal tau accumulation is an early feature of the injury [21]. Late neuroinflammation (initially perivascular) and synaptic loss appear together with neurobehavioral alterations) [31, 33, this paper]. The resulting PTSD-like phenotype has at least in part a glutamatergic basis as it is rescued with a metabotropic glutamate receptor 2/3 antagonist [65]

Classical complement cascade genes are upregulated in astrocytes that induce synaptic damage (A1 astrocytes) [78]. A1 astrocytes lose their capacity to promote neuronal survival, outgrowth and synaptogenesis. They also lose the ability to phagocytize synaptic debris and myelin and they induce neuronal and oligodendrocytic death. By contrast, A2 activated astrocytes, induced by ischemia, have the potential to up-regulate many neurotrophic factors and thrombospondins, which favor neuronal growth and survival and promote synapse repair. Therefore, the balance between these activated populations of astrocytes can predict the course of the blast-induced disease [25, 68]. Figure 20 illustrates a hypothetical scheme for how the elements discussed above may be related to the pathophysiology of injury in this model.

### Blast-induced mechanical tears from superficial pial vessels induce the formation of arteriovenous-like formations

We have previously reported that low-level blast overpressures induce tears in the brains of blast-exposed rats [28]. These lesions appear to follow the fault lines of penetrating cortical vessels [28]. Initial blast-induced damage can occur through direct cranial transmission of blast waves or via thoracoabdominal vascular hydrodynamic mechanisms whereby a blast wave striking the body causes indirect CNS injury through what has been referred to as a thoracic effect [6, 24, 75]. In the example shown in Fig. 16, a tear expanded through the insular cortex and repositioned the sheared tissue. Such a tear should have been induced by an exterior superficial large vessel running longitudinally in a rostro-caudal direction. Based on the anatomical location of the insular lesion presented, longitudinal superficial branches of the middle cerebral artery or most likely the caudal rhinal vein may be candidates for inducing the observed tissue damage. In a manner homologous to whip physics [36], blast mechanical energy may induce a traveling loop through the vasculature that gains speed until it reaches the speed of sound, generating a sonic shock wave that tears through the tissue. Blast mechanical energy traveling through the vascular loop may initially affect the normal astrocytic coverage of the vasculature, triggering the observed acute and subacute astrocytic degenerative processes. Also, as shown in Figs. 16 and 17, tissue shearing also can result in the displacement and repositioning of perpendicularly-running vasculature deeper into the internal parenchyma.

The newly repositioned vascular cluster now deep within the brain parenchyma will in turn result in the formation of arteriovenous-like structures. Arteriovenous formations comprise tangled masses of arteries and veins with abnormal communicating channels between them, which disrupt normal blood flow by bypassing the capillary network. The continued high-pressured blood flow through the arteriovenous formations will eventually induce vascular structural changes that may lead to the formation of aneurysms that can rupture causing hemorrhages into the surrounding brain tissue. In humans, local traumatic injuries can lead to their development as a result of vascular structural weaknesses [37, 53, 85]. Our results provide evidence for a direct link between the formation of arteriovenous-like structures and blast-induced vascular-mediated tears. This pathology may occur with some frequency, as we identified an additional animal which had an arteriovenous malformations in the insular cortex. We previously described the presence of external pial vasculature deep within repositioned tissue in the insular cortex of a blast-exposed animal (see Fig. 7 in [28]).

The NOR test, which is used to evaluate recognition memory, has been very useful in the identification of altered behaviors in blast-exposed animals (Fig. 1) [64]. Object recognition (OR) is known to be influenced by insular cortical activity. Basolateral amygdala activation enhances OR memory by inhibiting anterior insular cortex activity [12]. Moreover, it is known that c-fos expression is induced in the hippocampus (CA1 and CA3 regions), insular cortex, perirhinal cortex, and medial prefrontal cortex when object recognition memory is generated, suggesting that gene expression in these brain regions contributes to the formation of OR memory [83]. Overall, our results provide further evidence that blast-induced lesions in these brain regions and/or in the vasculature involved in the irrigation of these areas may result in the development of the characteristic PTSD-like phenotype.

### Laterality of the brain response

In our model, blast exposures are delivered frontally and there should be no systematic variation in the rat's placement within the blast tube that would cause the right or left hemisphere to be differentially impacted. Yet, in this study as in previous studies, we have noted prominent laterality effects [21, 63]. For example, tau is preferentially hyperphosphorylated in the right hemisphere compared to the left following blast exposure, changes that could be correlated with behavior [21, 63]. Thus, we were not surprised to find differences in vascular pathology or inflammation between the hemispheres. We can only speculate that this pattern reflects some functional and structural hemispheric laterality in the rat, which causes the hemispheres to be differently affected by the shock wave. As with handedness in humans, rats and mice are known to exhibit paw preference and hemispheric laterality for complex behavioral functions [19]. Hemispheric dominance has been in particular found to affect spatial memory in rodents [46, 74] and behavioral asymmetries have been correlated with biochemical asymmetries in rodent brain, particularly in the dopaminergic system [80]. Others have also noted asymmetric responses to focal cortical injuries which may have a neurohormonal basis [4]. Future studies will be needed to explore this interesting aspect of blast injury.

### Study limitations

One limitation of the present study is the lack of inclusion of female rats. Sex differences in outcomes after TBI are well known [38]. Studies indicate that female veterans are more likely to report persisting neurobehavioral symptoms and use more outpatient services than their male counterparts [13]. Sex differences in response to blast exposure have been scarcely studied, although two recent reports have suggested that blast responses in female rats may differ [40, 57]. With the increasing number of female veterans these studies assume a high importance. Housing conditions might also affect expression of the blast-induced behavioral phenotype. Having originally found that a blast-induced chronic phenotype was present in singly housed males [22], it has been difficult to move away from single housing for chronic studies such as those described here. In male rats, single housing avoids dominance order effects and fighting. The equal single housing conditions of blast and sham-exposed animals in this study, should limit any interaction effects.

## Conclusions

Overwhelming evidence indicates that diverse cerebral vascular degenerative processes arise from blast overpressure exposures. Mechanisms for blast-induced vascular degeneration include alterations in the neurovascular unit through the loss and degeneration of perivascular astrocytes; endothelial, intimal and adventitial alterations (including neointima formation and ECM remodeling); and formation of arteriovenous structures associated with blast-induced tissue tears. These vascular alterations result in structural changes, leading to vascular leakage, tissue hypoperfusion and likely deficiencies in the glymphatic cerebrospinal fluid flow and intramural interstitial spinal fluid periarterial drainage. These events can generate a cascade of events leading to neuroinflammation with the consequent neuronal dysfunction and loss.

## Data Availability

The datasets generated during and/or analysed during the current study are available from the corresponding author on reasonable request.
